# Alzheimer’s disease-associated complement gene variants influence plasma complement protein levels

**DOI:** 10.1186/s12974-023-02850-6

**Published:** 2023-07-21

**Authors:** Aurora Veteleanu, Joshua Stevenson-Hoare, Samuel Keat, Nikoleta Daskoulidou, Henrik Zetterberg, Amanda Heslegrave, Valentina Escott-Price, Julie Williams, Rebecca Sims, Wioleta M. Zelek, Sarah M. Carpanini, Bryan Paul Morgan

**Affiliations:** 1 UK Dementia Research Institute Cardiff, School of Medicine, Cardiff University, Hadyn Ellis Building, Maindy Road, Cardiff, CF24 4HQ UK; 2 Neurosciences and Mental Health Institute, Cardiff University, Cardiff, CF244HQ UK; 3 UK Dementia Research Institute at University College London, London, WC1E6BT UK; 4 Department of Psychiatry and Neurochemistry, Institute of Neuroscience and Psychology, The Sahlgrenska Academy at the University of Gothenburg, Mölndal, Sweden; 5 Clinical Neurochemistry Laboratory, Sahlgrenska University Hospital, Mölndal, Sweden; 6 Department of Neurodegenerative Disease, UCL Institute of Neurology, Queen Square, London, WC1N3BG UK; 7 Hong Kong Center for Neurodegenerative Diseases, Clear Water Bay, Hong Kong, China; 8 Division of Psychological Medicine and Clinical Neurosciences, Cardiff University, Cardiff, CF244HQ UK

**Keywords:** Alzheimer’s disease, Complement system, Biomarkers, Clusterin, Complement receptor 1, C1s, C1q, Factor H, GWAS

## Abstract

**Background:**

Alzheimer’s disease (AD) has been associated with immune dysregulation in biomarker and genome-wide association studies (GWAS). GWAS hits include the genes encoding complement regulators clusterin (*CLU*) and complement receptor 1 (*CR1*), recognised as key players in AD pathology, and complement proteins have been proposed as biomarkers.

**Main body:**

To address whether changes in plasma complement protein levels in AD relate to AD-associated complement gene variants we first measured relevant plasma complement proteins (clusterin, C1q, C1s, CR1, factor H) in a large cohort comprising early onset AD (EOAD; n = 912), late onset AD (LOAD; n = 492) and control (n = 504) donors. Clusterin and C1q were significantly increased (p < 0.001) and sCR1 and factor H reduced (p < 0.01) in AD plasma versus controls. ROC analyses were performed to assess utility of the measured complement biomarkers, alone or in combination with amyloid beta, in predicting AD. C1q was the most predictive single complement biomarker (AUC 0.655 LOAD, 0.601 EOAD); combining C1q with other complement or neurodegeneration makers through stepAIC-informed models improved predictive values slightly. Effects of GWS SNPs (rs6656401, rs6691117 in *CR1*; rs11136000, rs9331888 in *CLU*; rs3919533 in *C1S*) on protein concentrations were assessed by comparing protein levels in carriers of the minor vs major allele. To identify new associations between SNPs and changes in plasma protein levels, we performed a GWAS combining genotyping data in the cohort with complement protein levels as endophenotype. SNPs in *CR1* (rs6656401), *C1S* (rs3919533) and *CFH* (rs6664877) reached significance and influenced plasma levels of the corresponding protein, whereas SNPs in *CLU* did not influence clusterin levels.

**Conclusion:**

Complement dysregulation is evident in AD and may contribute to pathology. AD-associated SNPs in *CR1*, *C1S* and *CFH* impact plasma levels of the encoded proteins, suggesting a mechanism for impact on disease risk.

**Supplementary Information:**

The online version contains supplementary material available at 10.1186/s12974-023-02850-6.

## Background

Alzheimer’s disease is a progressive neurodegenerative disease and the most common cause of dementia, inflicting enormous personal, social, economic and societal costs [1]. Classical AD pathology comprises amyloid beta (Aβ) plaque deposition and hyperphosphorylated tau (p-tau) tangle accumulation [2], although Aβ plaques are frequent in brains of cognitively unimpaired elderly individuals [3]. Early diagnosis, essential for effective interventions, is difficult. Genetic, behavioural, imaging and fluid biomarker methods have all been proposed. In cerebrospinal fluid (CSF), levels of the Aβ_42_ and Aβ_40_ peptides, total or hyperphosphorylated tau (tTau or pTau), neurofilament light (NfL; nonspecific marker of neuronal damage) and glial fibrillary acidic protein (GFAP; marker of astrocytic reactivity) collectively establish the AT(N) (amyloid/tau/neurodegeneration) diagnostic framework for AD, currently the best predictive biomarker set [4]. Given the invasive nature and cost of CSF collection and brain imaging, reliable blood biomarkers that facilitate AD diagnosis are much needed; plasma AT(N) biomarkers can be informative in predicting AD [5, 6], but other biomarkers are needed. AD genetics point to the immune system as a potential source.

Inflammation has long been recognised as a culprit in neurodegenerative diseases, with many chronic inflammatory conditions, including obesity, diabetes and inflammatory bowel disease, associated with increased risk for AD [7–9]. An intact blood brain barrier (BBB) ensures brain immune privilege; however, BBB disruption occurs in AD permitting immune mediators from the periphery to penetrate the brain parenchyma and cause brain inflammation. Neuroinflammation markers, including pro-inflammatory cytokines [10], have been proposed as biomarkers for neurodegenerative diseases. Complement is a core part of the innate immune system and potent driver of inflammation in immune defence and in pathology. Evidence implicating complement in AD emerged in the 1980s; immunostaining of brain tissue showed C1q, C3, factor H (FH), and clusterin colocalising with Aβ plaques and surrounding sites of neuronal damage [11, 12]. Moreover, aggregated Aβ directly activated the complement cascade by interacting with C1q [13], and C1s displayed chaperone activity to inhibit aggregation of Aβ_1–42_ fibrils in vitro [14]. Many studies of complement proteins as AD biomarkers in plasma and/or CSF have been published with inconsistent results, although plasma clusterin consistently emerges as a biomarker for AD [15, 16].

Sporadic AD shows a significant contribution from genetics, accounting for 68–79% heritability for late onset (after 65 years) AD (LOAD), rising to over 90% for those with onset before 65 (early onset; EOAD) [17]. Seventy-five genome-wide significant AD risk loci have been identified to date [18]; these include the complement genes *CLU* and *CR1*, both in the top 5 most significant hits in GWAS [19], and recently a suggestive association with *C1S* (OR 1.05, p = 9.9 × 10^–7^) [18]. The risk variants in these genes are single nucleotide polymorphisms (SNPs) in non-coding regions (rs11136000, rs9331888 in *CLU*; rs6656401 in *CR1*; rs3919533 in *C1S*), or within exonic regions, causing amino acid substitutions (rs6691117 in *CR1;* I2065V). The *CLU* minor allele at rs11136000 is associated with reduced AD risk [20]. AD risk SNPs in *CR1* are associated with *CR1* length polymorphism; the minor allele at rs6656401 marks carriers of the CR1*2 isoform expressing an additional long homologous repeat and C3b binding site [21]. Initially identified as risk for age related macular degeneration (AMD), SNPs in *FH* have been associated with rate of cognitive decline in AD and shown to modify *FH* mRNA expression in the brain, leading to impaired complement regulation [22, 23]. For other non-coding SNPs in complement genes, mechanism of effect is not known.

While previous large genetic studies have performed GWAS to identify new loci associated with AD, GWAS using an intermediate phenotype (including plasma or CSF protein levels, imaging data, or any other quantifiable trait) allow a deeper analysis of uncharacterised mechanisms of effect [24–28]. The endophenotype more directly interrogates the effect of a single gene, is genetically simpler, evaluates the direct effect of the SNP and is less impacted by other genetic and biological variables than conventional GWAS. The resultant reduction in “noise” increases statistical power enabling the use of smaller cohorts. Using protein biomarker concentrations as endophenotype permits interpretation of the role of GWAS-implicated intronic SNPs by linking them directly to a change in plasma levels. This approach can also identify novel SNPs that influence the expression of a protein of interest and increase statistical power by focusing on a single quantitative trait [29, 30]. Although our focus was on AD GWAS-implicated SNPs in complement genes, the endophenotype approach enables additional complex genetic analysis to screen for all genomic loci that associate with changes in plasma levels of the complement biomarkers, critical because protein expression is determined by a multi-locus consensus involving coding and non-coding regions.

We measured five complement biomarkers, clusterin, soluble CR1 (sCR1), C1s, C1q and FH, selected based on genetic or functional association with AD, in a cohort comprising 504 cognitively unimpaired elderly controls, 912 EOAD and 492 LOAD subjects. Predictive value of individual complement biomarkers and sets of complement biomarkers in combination with AT(N) markers was assessed in ROC analysis. Complement biomarker levels were then used as endophenotype in a GWAS to identify SNPs that impacted complement biomarker levels.

## Main body

### Methods

#### Subjects

Sporadic AD and control plasma samples [n = 504 control, 912 EOAD (onset < 65 years), 492 LOAD (onset > 65 years)] were a subset of the AD Cardiff Cohort, collected between 2004 and 2020 from individuals recruited from UK community and hospital settings using MRC, Moondance Foundation, and Health and Care Research Wales (HCRW) funding (Table 1). All individuals were of Caucasian descent. The effect of storage time was tested and found not to significantly affect concentrations of the measured proteins.

**Table 1:** Cohort clinical, demographic, and genetic information

	EOAD	LOAD	Control
Number	912	492	504
Sex, n (%)			
Female	425 (46.6)	243 (49.39)	282 (56)
Male	487	249	222
Age at onset			
Mean (SD)	58 (5.16)	71 (5.34)	n/a
Range	28–65	66–90	n/a
Age at inclusion^a^			
Mean (SD)	64 (5.7)	76 (5.46)	82.5 (6.8)
Range	30–90	66–97	59–100
Disease duration (SD)	5.7 (3.6)	4.5 (2.9)	n/a
MMSE score (SD)	16.34 (10)	18 (9.23)	27.4 (5.5)
*APOE* status, n (%)			
ε4 (−)	402	184	400
ε4 (+)	491 (55)	289 (61)	93 (18.9)
*CLU* rs11136000, n (%) (MAF_A = 0.3659, MAF_U = 0.4)			
CC	370 (40.57)	204 (41.46)	172 (34.68)
TC	412 (45.18)	227 (46.14)	242 (48.79)
TT	130 (14.25)	61 (12.4)	82 (16.53)
*CLU* rs9331888, n (%) (MAF_A = 0.29, MAF_U = 0.28)			
CC	462 (50.77)	236 (51.42)	254 (51.84)
GC	374 (41.1)	177 (38.56)	201 (41.02)
GG	74 (8.13)	46 (10.02)	35 (7.14)
*CR1* rs6656401, n (%) (MAF_A = 0.1958, MAF_U = 0.1735)			
GG	596 (65.49)	290 (63.18)	336 (68.71)
AG	285 (31.32)	145 (31.59)	137 (28.02)
AA	29 (3.19)	24 (5.23)	16 (3.27)
*CR1* rs6691117, n (%) (MAF_A = 0.1822, MAF_U = 0.1792)			
AA	593 (65.02)	332 (67.48)	337 (68.08)
GA	299 (32.79)	148 (30.08)	144 (29.09)
GG	20 (2.19)	12 (2.44)	14 (2.83)
*C1S* rs3919533, n (%) (MAF_A = 0.1545, MAF_U = 0.1667)			
TT	659 (72.26)	336 (68.43)	345 (68.86)
CT	237 (25.99)	146 (29.74)	138 (27.54)
CC	16 (1.75)	9 (1.83)	18 (3.6)
*CFH* rs6664877, n (%) (MAF_A = 0.175, MAF_U = 0.1549)			
CC	575 (65.64)	309 (69.44)	313 (71.62)
TC	286 (32.65)	124 (27.87)	114 (26.1)
TT	15 (1.71)	12 (2.69)	10 (2.28)

*EOAD* early onset AD, *LOAD* late onset AD, *MMSE* mini mental state examination, *MAF_A* minor allele frequency in individuals with AD, *MAF_U* minor allele frequency in control individuals

^a^EOAD and LOAD subjects were significantly younger than control subjects (p < 0.0001)

AD diagnosis was established using a comprehensive, standardized and validated clinical and neuropsychological assessment [31], in accordance with the National Institute of Neurological and Communication Disorders and Stroke and the Alzheimer's disease and Related Disorders Associations (NINCDS-ADRDA) clinical diagnostic criteria for AD. All diagnoses were based on a semi-structured interview with known validity for AD pathology (i.e. positive predictive value of 92–95%) which included: mini-mental state examination (MMSE); The Cambridge Mental Disorders of the Elderly Examination (CAMDEX; informant interview); The Blessed Dementia Scale; The Bristol Activities of Daily Living Scale; Webster Rating Scale; Global Deterioration Scale (GDS); Cornell Scale for Depression in Dementia; Neuropsychiatric Inventory (NPI) (12-item version) [31, 32]. Control subjects were either spouses of AD patients or selected from primary-care practices situated in the same geographical areas. Assessment of controls was as described above, including CAMDEX and GDS; exclusion criteria were the presence of dementia, depression, delirium, or other illnesses likely to significantly reduce cognitive function. Controls were purposely selected for advanced age (age range 59–100; mean 82.5) with no dementia to reduce the potential for conversion to disease [33]. Age at assessment, sex, MMSE score, and genome-wide array genotyping (Illumina 610, Illumina 550, or global screening array) was available for most samples. Age at onset was also available for 1396 cases, and disease duration was calculated for these. Ethical approval was obtained from the Multi-centre Research Ethics Committee, relevant local ethics committees and NHS trusts in the recruiting regions. Demographics are summarised in Table 1.

#### Measurement of complement proteins by ELISA

Five complement components (clusterin, sCR1, C1s, C1q, FH) were measured in all plasma samples by ELISA. Antibodies, protein standards, and assay characteristics are detailed in Additional file 1: Table S1. Plasma samples stored at − 80 °C were defrosted immediately prior to assay, vortexed briefly, diluted in phosphate-buffered saline containing 0.05% Tween-20 (PBST) and 0.2% bovine serum albumin (BSA) and kept on ice or stored at − 80 °C until used.

Capture antibodies were immobilised overnight at 4 °C on 96-well immunoplates (Fisher Scientific #1039451) at concentrations between 2–20 µg/ml in 50 µl/well carbonate-bicarbonate buffer (pH 9.6). Wells were blocked by incubation with 100 µl 2% BSA in 0.05% PBST for 1 h at 37 °C, washed once with PBST, and plasma samples or protein standards (50 µl) added at a suitable dilution (Additional file 1: Table S1). Plates were incubated for 90 min at 37 °C, washed three times and detection antibodies added at concentrations between 1–2 µg/ml in 50 µl/well 0.2% BSA in PBST for 1 h at 37 °C. For assays where the detection antibody was not directly labelled, HRP-labelled secondary antibody (anti-mouse or anti-rabbit IgG as appropriate, Jackson ImmunoResearch #715-035-151, #711-035-152) was added to washed plates at a suitable dilution for 1 h at 37 °C. Finally, plates were washed and developed using OPD substrate (Sigma-Aldrich, #P9187) for 3–15 min (consistently for each assay), followed by addition of 5% H_2_SO_4_ to quench the reaction. Optical densities were read at 492 nm using a microplate reader (Infinite F50, Tecan #30190077). All samples were measured in duplicate, blinded to diagnosis. Intra- and inter-assay coefficients of variation were below 15% for all assays.

#### Measurement of p-tau181, Aβ40, Aβ42, NfL, GFAP

Plasma concentrations of p-tau181, Aβ40, Aβ42, NfL and GFAP had previously been measured in these samples using Simoa assays (Quanterix, Billerica, MA, USA) [34]. The measurements were performed in one round of experiments using one batch of reagents with the analysts blinded to diagnosis and clinical data. Intra-assay coefficients of variation were below 10%.

#### Statistics

Data were analysed by constructing an 8-point standard curve using known concentrations of pure protein for each assay, interpolating the averaged optical density values for each sample on the curve, and multiplying the obtained values by the dilution factor. Data were plotted using GraphPad Prism 5, tested for normality using the Kolmogorov–Smirnov test and found not to be normally distributed. Data were analysed statistically (α = 0.05) using IBM SPSS Statistics 26 by Mann–Whitney, Kruskal–Wallis tests with Dunn’s multiple comparisons post-hoc test, generalised linear models adjusting for sex and age (formula: Protein ~ Age + Sex + Phenotype) or Pearson correlations as appropriate. To test the effect of SNPs on protein levels, an interaction term between SNP and disease was used (formula: Protein ~ Age + Sex + Phenotype*SNP).

For ROC analyses, a series of generalised linear models (GLMs) using different combinations of protein measurement data were constructed in R using the base “stats” package, with a “binomial” model for error distribution and link function specified. EOAD and LOAD phenotypes were separated and classified as “1” for EOAD, “2” for LOAD and “0” for controls. The GLMs followed the formula pattern: Phenotype ~ Protein 1 (+ Protein 2…). A stepAIC (Akaike Information Criterion) model was run for both EOAD and LOAD to identify the optimal features to retain in the final model. To enhance the generalisability and applicability of our approach, models with fewer protein measurements were favoured. Data were randomly split 70:30 into “training” and “test” sets to prevent over-fitting and stratified to maintain case/control proportions; area under the curve (AUC) of the “test” data in ROC analysis was reported. Prior to analysis, protein levels were adjusted for age and sex and standardised to a mean of 0 and standard deviation of 1 to maintain equal contributions of each protein to analyses and prevent bias from proteins with wider ranges; both unadjusted and adjusted values were tested in the ROC analysis. The different models were compared using ROC curves; 95% confidence intervals were calculated using the default ‘bootstrap’ method with 2000 replicates for each AUC to provide a measure of uncertainty and model stability.

#### GWAS

All individuals included in the analysis had both genetic and biomarker information available. Genotype data were quality controlled (QCed) as described previously [35] and imputed via the Michigan Imputation Server using Minimac3 [36] and the Haplotype Reference Consortium reference panel. The data were combined and QCed with heterozygosity abs(F-het) > 5%, missing data proportion per person > 5%, related individuals with $\hat{\pi }$ > 0.2, gender mismatch, or were population outliers based on European population from 1000 Genomes [37]. Variants with minor allele frequency (MAF) < 1%, missing data proportion (MISS) > 5%, or Hardy–Weinberg Equilibrium (HWE) p ≤ 10^–6^ were excluded. To exclude batch effects, an association test was run between controls and variants; those variants with p-value < 0.001 were excluded, retaining 480,021 variants. Genetic data were aligned to human genome assembly GRCh37/hg19 and imputed as described above. Finally, all data were combined, related individuals with $\hat{\pi }$ > 0.2 were removed, and variants with MAF < 5%, poor accuracy of imputation (INFO) < 0.8, MISS > 5% or HWE p ≤ 10^–6^ were removed. After these corrections the final dataset contained 4,618,496 variants.

Genome-wide SNP-based association analyses were performed for each complement biomarker using linear regression modelling with PLINK as previously described [34]. Association analyses of SNPs with the biomarkers were adjusted for age and sex, five principal components (PCs) and case–control status (“caseness”), the latter introduced to reduce the variation due to differences in association patterns of biomarkers between cases and controls while retaining all available samples in the analysis to maintain statistical power. Genetic data for index SNPs associated with AD in *CLU, CR1, C1S,* and *APOE* (rs7412, rs429358) were extracted using PLINK, and LD between them determined using PLINK (https://www.cog-genomics.org/plink/2.0/, [38]). Manhattan plots were generated using qqman library in R, and association results for a particular gene/region were visualised using LocusZoom online tool (http://locuszoom.org, [39]). Raw GWAS data were uploaded to LocusZoom and set for PLINK analysis, the SNP of interest was specified under “region to display” with a ± 50–200 Kb flanking size, and R-square was determined in relation to the specified SNP, or the most significant SNP for *CFH*. R^2^ scores were calculated using the hg19/1000 Genomes Nov 2014 EUR dataset.

## Results

### Levels of complement proteins in plasma are altered in AD

**Fig. 1:**
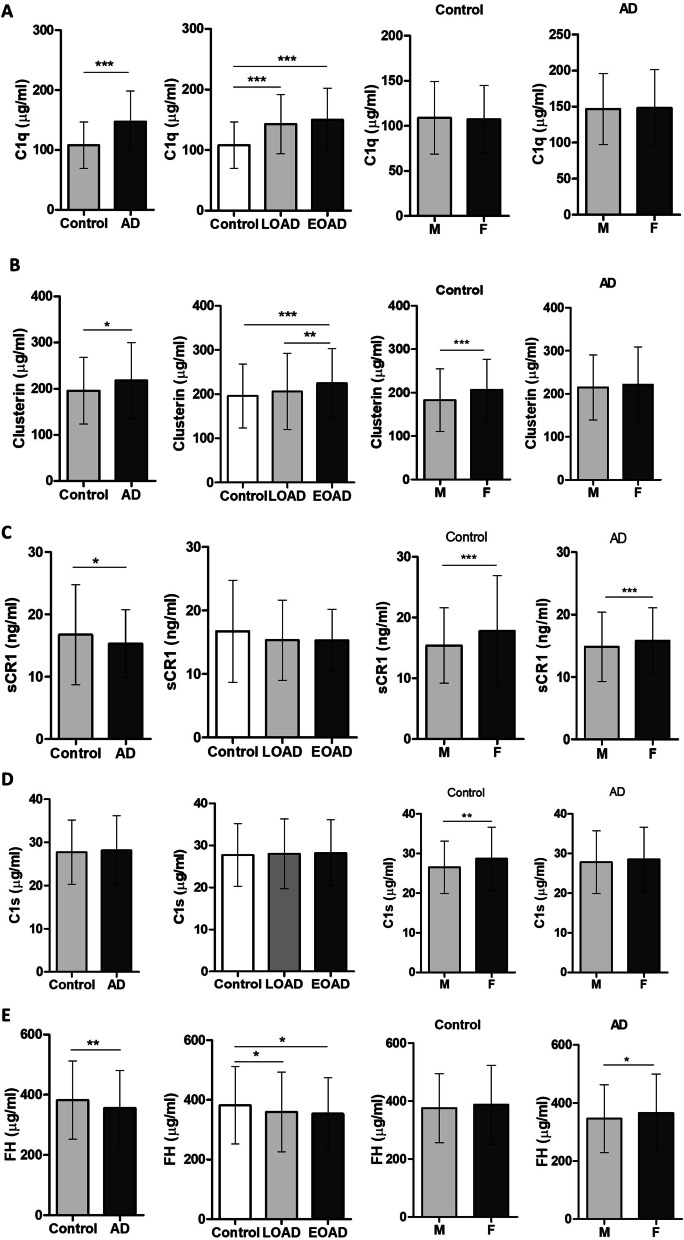
Complement proteins in AD. **A** C1q plasma levels were significantly elevated in both early and late onset AD compared to controls; there were no significant gender differences in C1q levels. **B** Clusterin levels were significantly elevated in early and late onset AD compared to controls; levels were significantly higher in females compared to males in controls but not AD. **C** Soluble CR1 levels were significantly decreased in AD compared to controls though significance was lost when split into early and late onset AD groups; levels were significantly lower in male subjects in AD and control groups. **D** Plasma C1s levels did not differ significantly between AD and controls; levels were significantly lower in males compared to females in the control group. **E** FH levels were significantly decreased in both early and late onset AD compared with controls; levels were significantly lower in males compared to females in the AD group. Data were corrected for age and sex; results are shown as mean ± SD, analysed using Mann–Whitney tests or generalised linear models including age and sex as covariates. *p < 0.05; **p < 0.01; ***p < 0.001. *M* male, *F* female. Numbers for each data set are shown in Table 1

We measured complement components C1q, clusterin, sCR1, C1s, and FH, selected because each has been implicated in AD by immunohistochemistry, biomarker analyses or genetics. Compared to controls, early and late AD cases had significantly higher levels of plasma C1q (149.8 µg/ml EOAD, 142.9 µg/ml LOAD, 107.9 µg/ml control; p < 0.001, Fig. 1A) and clusterin (224.6 µg/ml EOAD, 205.8 µg/ml LOAD, 195.8 µg/ml control, p < 0.005; Fig. 1B). In control but not AD subjects, clusterin levels were significantly lower in men compared to women (206.8 vs 182.8 µg/ml, p < 0.001; Fig. 1B). Compared to controls, early and late AD cases had significantly lower levels of plasma sCR1 (15.29 ng/ml EOAD, 15.32 ng/ml LOAD, 16.74 ng/ml control; p = 0.031, Fig. 1C) and FH (353.7 µg/ml EOAD, 359.7 µg/ml LOAD, 382.3 µg/ml control, p < 0.05, Fig. 1E). Plasma sCR1 levels were lower in males compared to females in AD and control samples (14.83 vs 15.82 ng/ml in AD, p < 0.001; 15.41 vs 17.82 ng/ml in control, p < 0.001, Fig. 1C). In AD cases, FH levels were significantly lower in men compared to women (346.3 vs 366.2 µg/ml, p < 0.01, Fig. 1E). Plasma levels of C1s did not differ between AD and control subjects (28.22 µg/ml EOAD, 28.02 µg/ml LOAD, 27.72 µg/ml control, Fig. 1D). In control but not AD samples, males had significantly lower levels of C1s compared to females (26.52 vs 28.71 µg/ml, p < 0.05, Fig. 1D).

### Complement protein levels correlate with each other and some AT(N) markers

To identify patterns of dysregulation in the complement system and relationships between complement and already established AD biomarkers, we performed correlation analyses. Significant positive correlations were identified between C1s/C1q, C1q/FH, C1s/FH in both AD and control groups (p < 0.001, Additional file 1: Table S2). No significant correlations were identified between complement protein levels and MMSE score, age at onset/study inclusion or disease duration. In controls, significant positive correlations were found between complement and AT(N) biomarker levels: clusterin/NfL; sCR1/GFAP, sCR1/NfL; C1q/Aβ40, while in the AD group there were significant negative correlations between clusterin/Aβ40, clusterin/Aβ42 (Additional file 1: Table S2).

### ROC analyses identify complement proteins that distinguish AD from controls

**Fig. 2:**
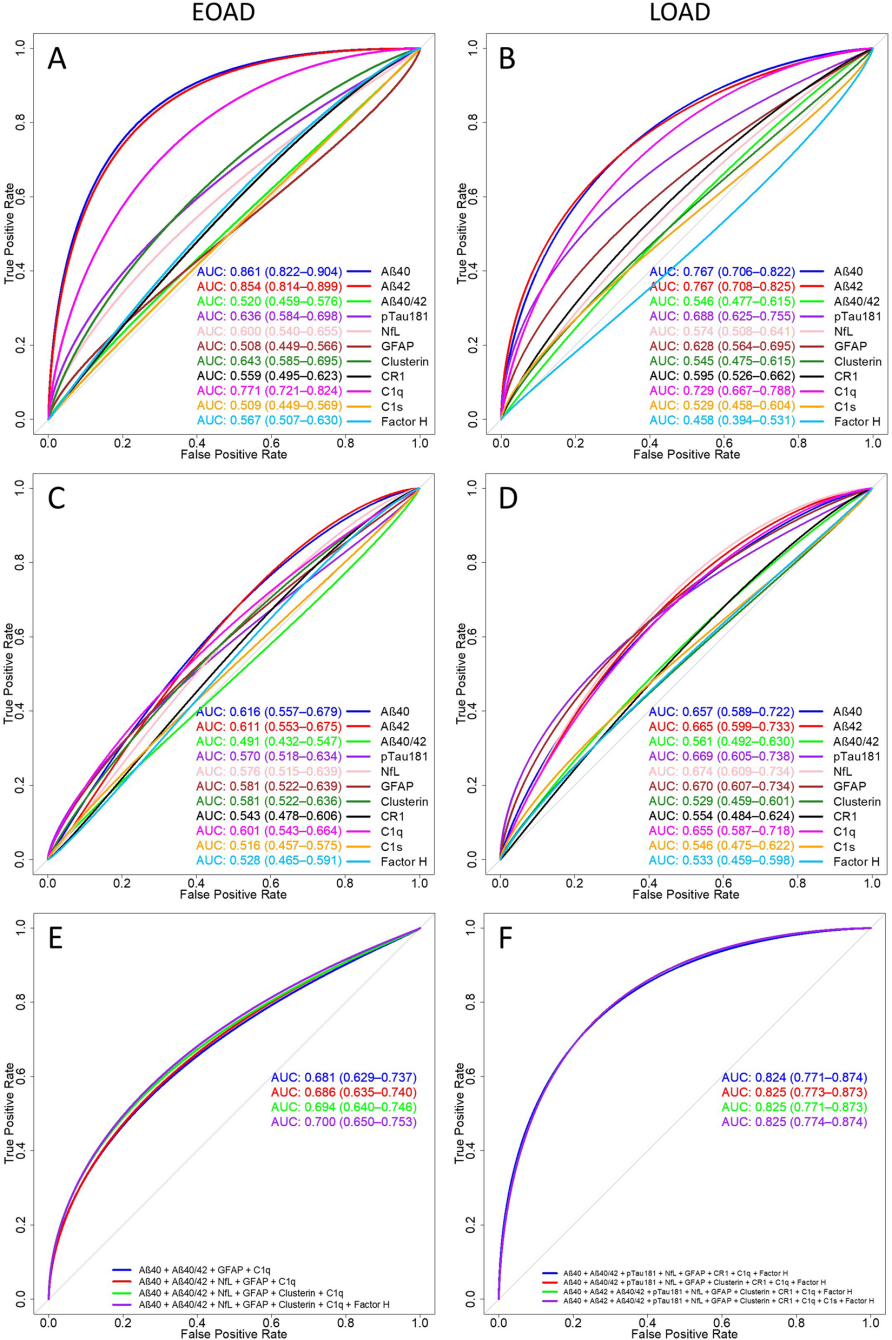
ROC Curves demonstrate the AD predictive capacity of complement biomarkers. ROC curves were generated using multiple GLMs for each protein. AUC statistics for individual (**A**–**D**) or combined (**E**, **F**) analytes are shown for EOAD (**A**, **C**, **E**) and LOAD (**B**, **D**, **F**). 95% confidence intervals, calculated using bootstrapping with 2000 replicates, are included in brackets for each GLM. Proteins unadjusted (**A**, **B**) and adjusted (**C**, **D**) for age and sex were included for comparison. Linear predictors were selected based on results of stepAIC models of adjusted data for EOAD (**E**) and LOAD (**F**), with the best model based on highest AICs relative to model complexity plotted first (EOAD = Aβ40 + Aβ40/Aβ42 + GFAP + C1q; LOAD = Aβ40 + Aβ40/Aβ42 + pTau18 + NfL + GFAP + CR1 + C1q + Factor H for LOAD) and then 3 regressive steps from the stepAIC results plotted sequentially

ROC analyses were performed on complement and AT(N) biomarkers to determine their utility in distinguishing LOAD and EOAD from control (Fig. 2). The AD groups were analysed separately because direction of effect for each protein (Additional file 1: Fig. S1) and distributions of protein concentrations across groups and ages (Additional file 1: Figs. S2–S3) differed significantly between LOAD and EOAD (Additional file 1: Table S3). Protein levels, unadjusted (Fig. 2A, B) and adjusted for age/sex (Fig. 2C, D) were included in ROC analyses for comparison. Among complement proteins analysed individually, C1q reached the highest AUC (0.601 for EOAD, 0.655 for LOAD), while amyloid markers performed best among AT(N) proteins (Aβ40 0.616 for EOAD, 0.657 for LOAD; Aβ42 0.611 for EOAD, 0.665 for LOAD; Fig. 2A–D); the Aβ42/Aβ40 ratio had a low AUC in both AD groups (0.491 EOAD, 0.561 LOAD). StepAIC models were used to identify best model for each phenotype. For EOAD, combining Aβ40, Aβ42/Aβ40, GFAP and C1q gave an AUC of 0.681 (AIC 1064.17); for LOAD, Aβ40, Aβ42/Aβ40, pTau181, NfL, GFAP, CR1, C1q, FH gave an AUC of 0.824 (AIC 644.41) (Additional file 1: Table S4). Inclusion of other biomarkers did not significantly improve performance (Fig. 2E, F). DeLong tests confirmed that levels of the implicated proteins had a significant effect on the predictive capacity of the overall model after accounting for age, sex and APOE status (Additional file 1: Table S5). Complete ROC statistics for the protein biomarker comparisons between EOAD and controls, LOAD and controls and EOAD and LOAD, both unadjusted and adjusted for age and sex are shown in Additional file 1: Tables S6 and S7.

### Clusterin levels are not impacted by AD risk SNPs or *APOE* status

**Fig. 3:**
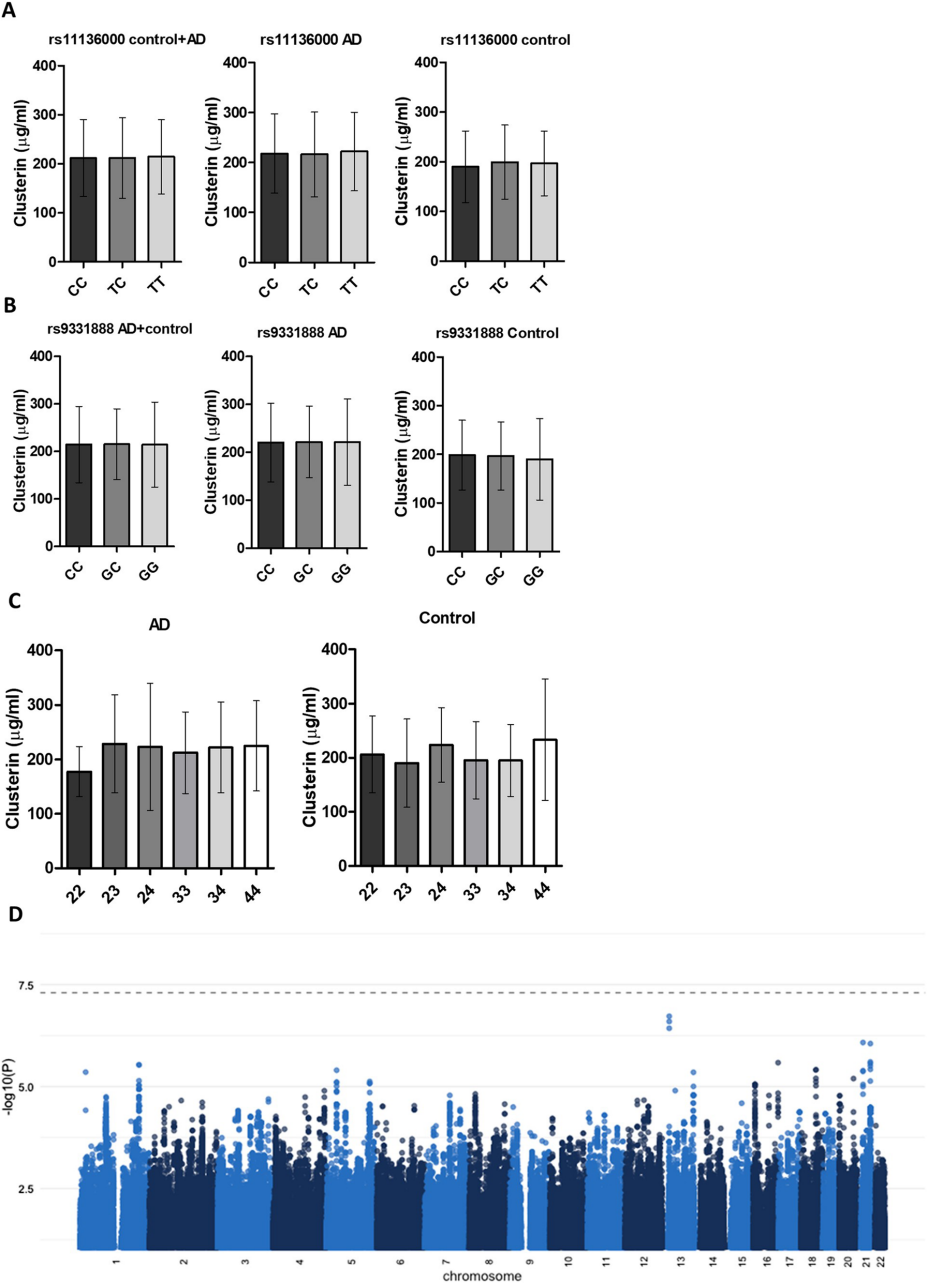
Effects of genetic variants in *CLU* on clusterin protein levels. **A**, **B** No significant effects of either rs11136000 (**A:** marking the LD block) or rs9331888 (**B**) in *CLU* were identified on plasma levels of clusterin). **C** Clusterin levels were not significantly different between *APOE* genotypes. Data are shown as mean ± SD and were analysed statistically using Kruskal–Wallis test with Dunn’s multiple comparisons post-hoc test. Numbers for homozygote and heterozygote carriers of each SNP are shown in Table 1. **D** Manhattan plot of GWAS results of the whole sample set using plasma clusterin levels as an intermediate phenotype found no significant variants

SNPs in *CLU* previously identified in AD GWAS [18] [rs11136000, rs9331896, rs2279590 in linkage disequilibrium (LD; r^2^ > 0.85) and rs9331888 (r^2^ with rs11136000 = 0.26)] were tested for impact on plasma clusterin by comparing levels in carriers of the minor alleles at rs11136000 (T/C, used as surrogate for SNPs in LD) and rs9331888 (G/C) with carriers of the major allele. Neither the SNP cluster defined by rs11136000 nor the unlinked SNP rs9331888 impacted clusterin levels, whether assessed in the whole sample set, AD alone or control alone (Fig. 3A, B). When using an interaction term between SNPs and disease status in a series of GLMs, no significant effects were found for either rs11136000 or rs9331888 on clusterin levels (Additional file 1: Table S8). There was no significant effect of *APOE* status on clusterin levels, although there was a non-significant trend towards increased levels in *APOEε4* carriers (Fig. 3C). To determine whether any other SNPs in the genome were associated with plasma clusterin levels, a GWAS was performed with plasma clusterin as endophenotype. The resulting Manhattan plot did not show any genetic variants in *CLU* or elsewhere in the genome that significantly associated with clusterin plasma levels (Fig. 3D).

### AD risk SNPs in *CR1* significantly impact sCR1 levels

**Fig. 4:**
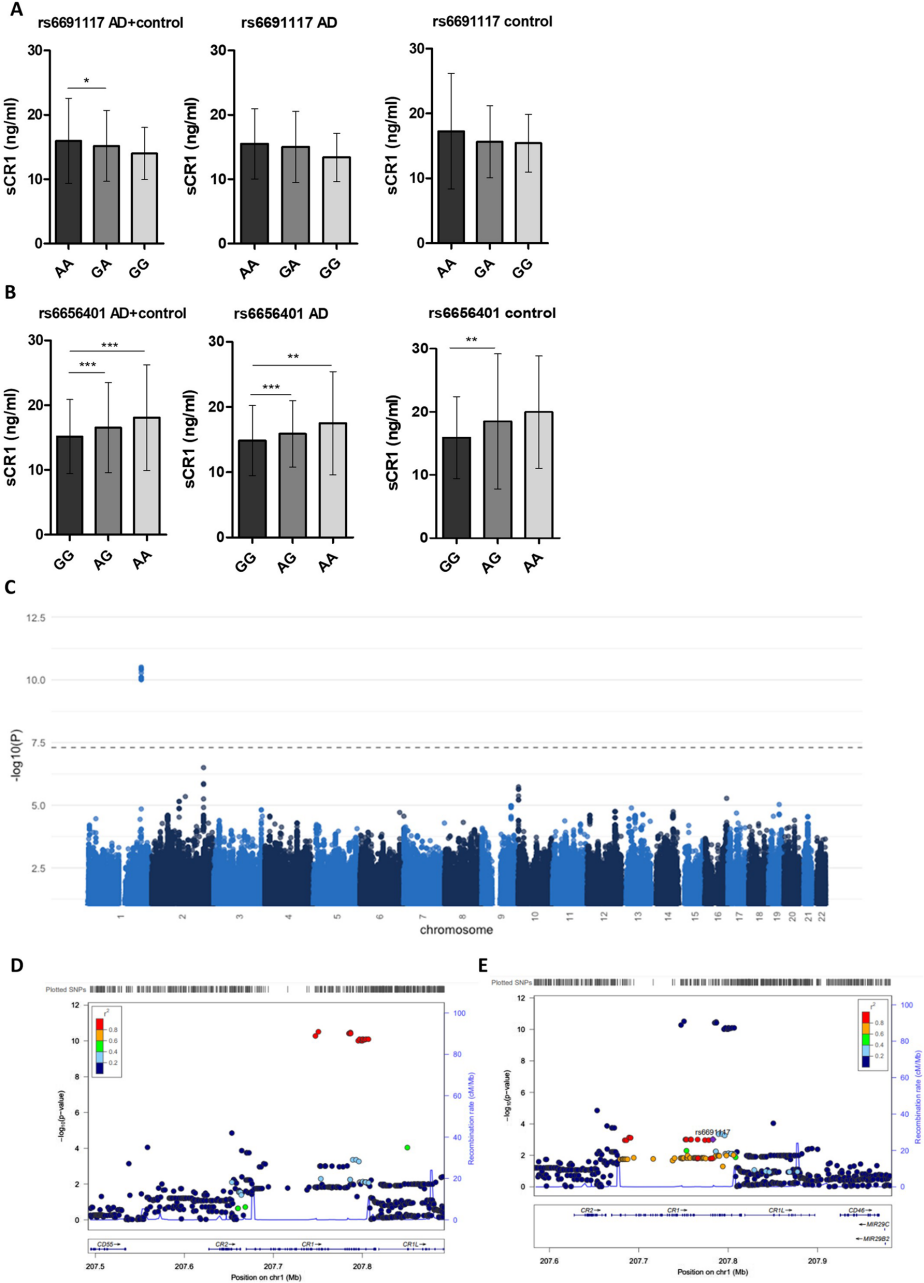
Effects of genetic variants in *CR1* on sCR1 protein levels. **A**, **B** The minor allele (G) at rs6691117 was significantly associated with a decrease in sCR1, while the minor (A) allele at rs6656401 was strongly associated with increased sCR1 levels in plasma. Data are shown as mean ± SD and were analysed using Kruskal–Wallis with Dunn’s multiple comparisons post-hoc tests. *p < 0.05; **p < 0.01; ***p < 0.001. Numbers for homozygote and heterozygote carriers of each SNP are shown in Table 1. **C** Manhattan plot of GWAS results on the whole sample set (n = 1667) with plasma sCR1 as endophenotype identifies multiple loci in the *CR1* gene on chromosome 1 significantly associated with changes in plasma sCR1 levels. **D**, **E** LocusZoom plots in the region identify a cluster of significant SNPs in high LD with rs6656401 (**D**), while rs6691117 was below the significance threshold (**E**)

The impact of AD-associated SNPs in *CR1* on plasma sCR1 levels was tested. Presence of the minor allele at rs6691117 (Ile2065Val, G/A) associated with significantly reduced plasma sCR1 levels in the combined cohort but when separated into AD and control groups the significance was lost (Fig. 4A). The intronic SNP rs6656401 (A/G) marks the AD-associated copy number variation in *CR1*; presence of the minor allele was associated with significantly higher sCR1 levels in the whole population and after separation into AD and control groups (Fig. 4B). An interaction term between SNPs and disease status in a series of GLMs revealed no significant effects for either rs6691117 or rs6656401 on plasma sCR1 levels (Additional file 1: Table S8). GWAS on the combined cohort identified a cluster of genome-wide significant loci in chromosome 1; LocusZoom views showed that these were within *CR1* in high LD with rs6656401, further strengthening the robust association between the rs6656401-tagged LD block and increased plasma sCR1 levels (Fig. 4D). In contrast, rs6691117 did not reach GWS for association with plasma sCR1 changes (Fig. 4E).

### C1s levels are significantly affected by SNPs in *C1S*

**Fig. 5:**
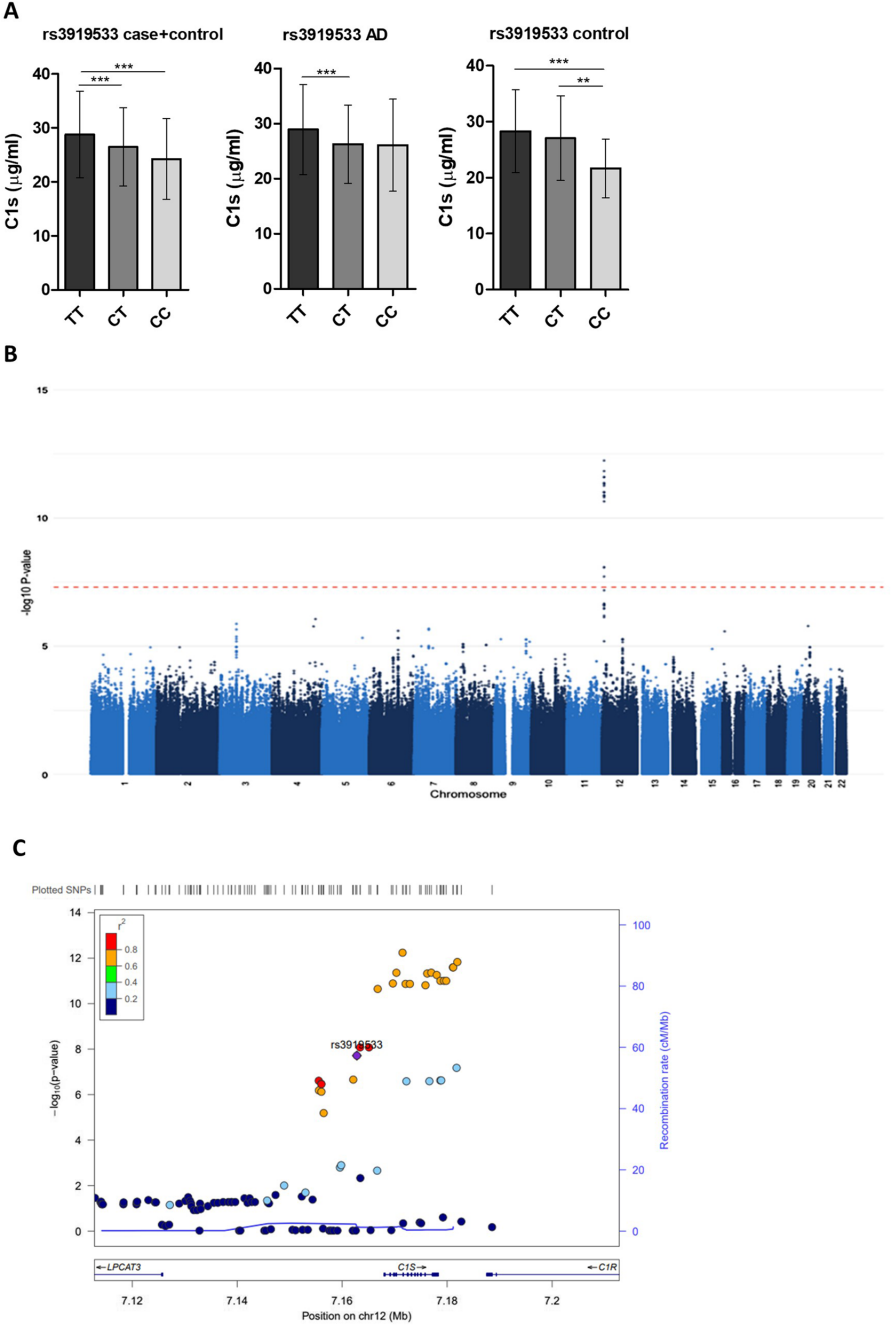
Effects of genetic variants in *C1S* on C1s protein levels. **A** Presence of the minor allele (**C**) at rs3919533 was gene dose-dependently associated with lower C1s levels in both the AD and control groups. Data are shown as mean ± SD and were analysed statistically using Kruskal–Wallis tests with Dunn’s multiple comparisons post-hoc test. **p < 0.01; ***p < 0.001. Numbers for homozygote and heterozygote carriers of the SNP are shown in Table 1. **B** Manhattan plot of GWAS results on the whole sample set (n = 1713) shows loci in chromosome 12 were significantly associated with changes in plasma C1s levels. **C** LocusZoom plot displays a cluster of significant SNPs within *C1S*, in moderate LD with rs3919533

Carriers of the minor allele at rs3919533 (C/T) in *C1S* had significantly lower C1s levels (Fig. 5A). GWAS performed on the combined cohort identified significant hits impacting C1s concentration in chromosome 12; LocusZoom views revealed a cluster of significant SNPs within *C1S* on chromosome 12 in moderate LD with the risk SNP rs3919533 located upstream of *C1S* (Fig. 5B, C).

### FH levels are significantly affected by SNPs in *CFH*

**Fig. 6:**
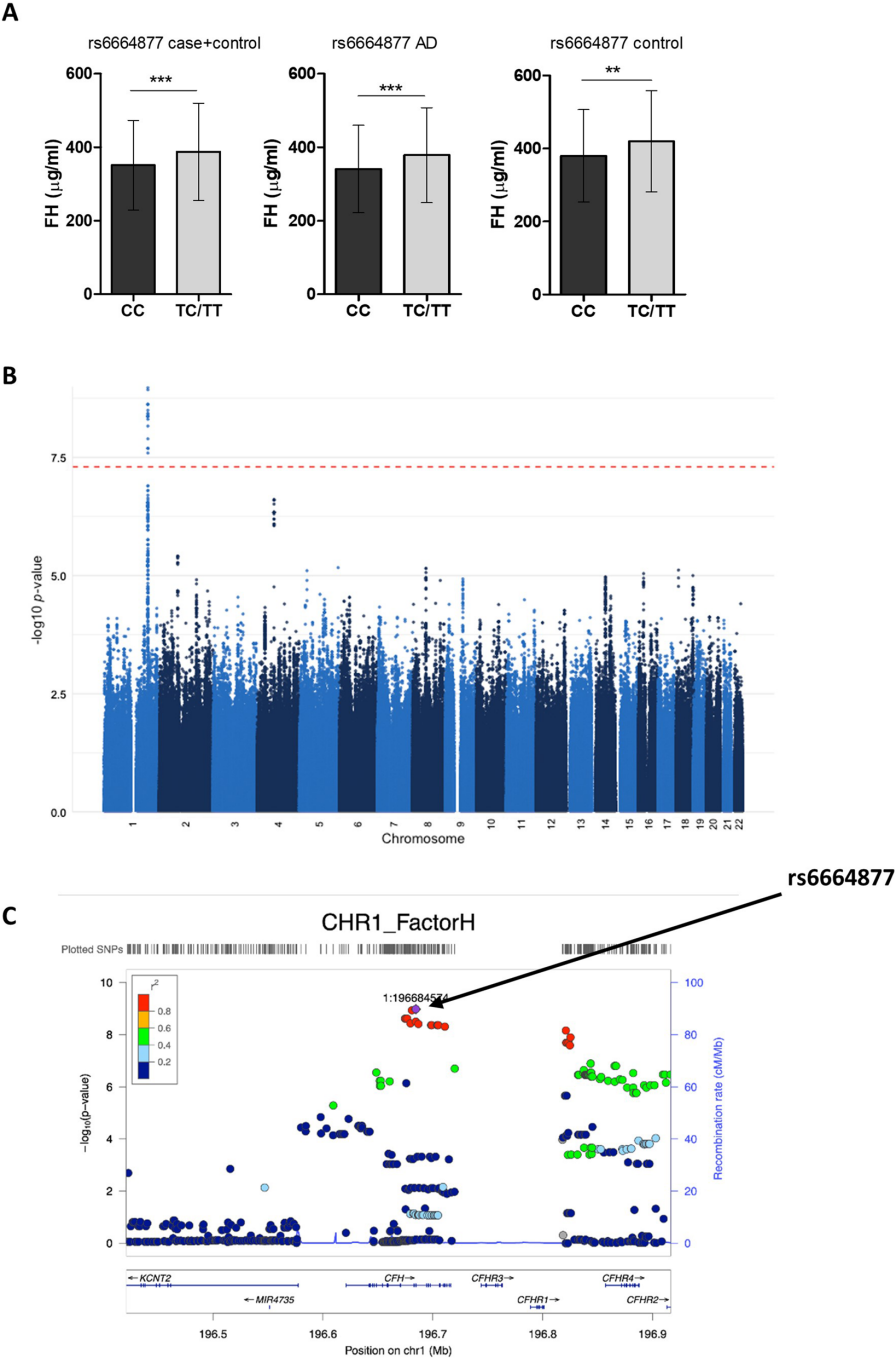
SNPs in *CFH* are significantly associated with changes in plasma FH levels. **A** Minor allele (T) carriers at rs6664877 had significantly higher plasma FH levels compared to major allele carriers in the combined cohort and in AD and controls analysed separately. Data are means ± SD and were analysed statistically using Mann–Whitney tests. **p < 0.01; ***p < 0.001. Numbers for homozygote and heterozygote carriers of the SNP are shown in Table 1. **B** Manhattan plot of GWAS results on the whole sample set (n = 1713) using plasma FH as endophenotype identifies loci in chromosome 1 significantly associated with changes in plasma FH levels. **C** LocusZoom analysis identifies a cluster of significant SNPs within *CFH* and a second cluster in the adjacent *CFHR4* gene that influence plasma FH levels. Rs6664877 was the most significant hit

The impact of AD-associated SNPs in *CFH* on plasma FH levels was tested. Carriers of the minor allele at rs6664877 (T/C) in *CFH* had a significant increase in FH levels in the combined cohort and after separation into AD and controls (Fig. 6A). GWAS performed on the combined cohort using plasma FH levels as endophenotype identified GWS hits in chromosome 1; LocusZoom views revealed clusters of significant SNPs within *CFH* and also in the adjacent *CFHR4* gene (Fig. 6B, C). Rs6664877 (T/C) in *CFH* was the most significant SNP impacting FH levels (p = 1.05 × 10^–9^).

## Discussion

As the incidence of AD climbs ever higher, there is a critical need for early detection, accurate diagnosis and prediction of disease risk. Predictive tests would enable population screening, close monitoring of at-risk individuals and pre-symptomatic intervention. Numerous studies have explored fluid biomarkers. The combination of CSF pTau, Aβ and neurofilament (AT(N)) aid diagnosis and are highly informative of disease progression, particularly useful in selection for and monitoring of clinical trials [5, 6]. However, CSF sampling is invasive and the high sensitivity assays required to measure the AT(N) markers in plasma are costly, restricting their broader use. This prompted us to seek blood biomarkers reflecting changes in the inflammatory component of AD. Complement has long been associated with AD; many studies have shown complement proteins, including C1q, clusterin, and FH, co-localising with Aβ plaques, and significant alterations in complement mRNA and protein levels in brain, plasma, and CSF in AD [12, 40]. Moreover, GWAS have consistently identified complement genes significantly associated with AD [18, 19].

We investigated plasma levels of AD-relevant complement proteins (C1q, clusterin, sCR1, C1s, FH) in AD patients and controls, and assessed their prediction accuracy in diagnosing AD alongside AT(N) biomarkers. C1q, the initiator of classical pathway activation, is present in amyloid plaques; in vitro, C1q enhanced Aβ aggregation but inhibited uptake of Aβ by microglia [41]. We found that plasma C1q was significantly increased in AD patients, suggesting increased complement activating capacity. C1q showed the best prediction accuracy among the measured complement proteins, particularly in late onset disease (AUC = 0.601 for EOAD, 0.655 for LOAD); adding in other complement biomarkers or AT(N) biomarkers did not increase the predictive accuracy of C1q alone. C1q levels are reported to increase with age and associate with age-related arterial stiffness [42]. The controls in our cohort were deliberately selected for advanced age; therefore, the observed differences cannot be explained by an age effect. C1q levels positively correlated with Aβ40 in controls, highlighting a subset of these who may be at increased risk for developing Aβ pathology.

Clusterin, also known as ApoJ, is the complement protein most studied as an AD biomarker. It is a multifaceted protein with important roles in AD pathology. It inhibits Aβ nucleation and enhances its clearance from the brain, and clusterin knockout mice develop Aβ deposition on cerebrovasculature [43–45]. Among its many roles, clusterin regulates the terminal pathway of complement, inhibiting formation of the membrane attack complex. Clusterin was previously reported to be increased in AD and mild cognitive impairment (MCI) plasma and CSF, although results are inconsistent [15, 46, 47]. In agreement with the consensus, we found a significant increase in plasma clusterin levels in AD patients, particularly in EOAD; however, this did not translate into good prediction accuracy in ROC analyses (AUC = 0.581 for EOAD, 0.529 for LOAD). Clusterin can readily cross the BBB, has been shown to sequester Aβ40 and prevent Aβ42 aggregation [14, 48–50]; the inverse correlation with plasma Aβ we identified may thus reflect ongoing changes in the AD brain. In controls, plasma levels of clusterin and NfL were positively correlated.

*CLU*, the gene encoding clusterin, is a major AD GWAS hit with multiple intronic SNPs identified, including a SNP cluster in tight LD (rs11136000, rs9331896, rs2279590) and an unlinked SNP (rs9331888) [19]. The SNP cluster associated with decreased AD risk and better cognitive scores [19, 20]; however, it was not associated with the increased *CLU* mRNA levels reported in AD brains [51]. GWAS using plasma clusterin as endophenotype revealed no significant associations between clusterin levels and variants in *CLU* or elsewhere in the genome, demonstrating that the observed differences in clusterin plasma levels were not caused by genetic variation in *CLU* or other genes included in current GWAS genotyping arrays. The report that clusterin levels were increased in brains but not plasma of *APOEε4* carriers [52–54] provoked us to test impact of *APOE* status on plasma clusterin levels; no significant differences were seen.

CR1, a receptor for the complement opsonic fragments C3b/C4b that plays key roles in immune complex handling in the periphery, was linked to AD in GWAS [19]. We recently showed that CR1 is abundantly expressed in brain, and that expression is markedly increased in AD [55]. Four isoforms of human *CR1* exist due to gene duplications and rearrangements, differing considerably in length. The two common forms, CR1*1 and CR1*2, comprise respectively 4 and 5 functional units termed long homologous repeats; the latter is risk for AD [21, 55]. In the current study, sCR1 was significantly decreased in AD patients, supporting our past findings in an independent cohort [56]; however, ROC analyses showed poor predictive accuracy for sCR1 (AUC = 0.543 for EOAD, 0.554 for LOAD). sCR1 levels correlated with NfL and GFAP levels in controls. SNPs in *CR1* have previously been shown to significantly contribute to AD risk and influence plasma sCR1 levels [21, 57]. The minor allele at rs6656401, associated with expression of the risk variant CR1*2 [21], was linked to accelerated cognitive decline [58], reduced CR1 density on erythrocytes and increased sCR1 plasma levels [59]. We also found that carriers of the rs6656401 SNP, whether AD or control, had significantly increased plasma sCR1 levels. In contrast, carriers of the minor allele at rs6691117, a missense variant (I2065V) associated with decreased brain volume in MCI subjects [60], had significantly decreased sCR1 levels; the membrane-proximal position of this amino acid change suggests an effect on proteolytic cleavage of CR1. The causative association between these SNPs and changes in plasma sCR1 levels was tested using endophenotype GWAS; the rs6656401-tagged LD block associated with expression of CR1*2 was highly significant in the analysis, confirming its direct effect on plasma sCR1 levels; in contrast, the rs6691117 SNP did not reach GWS for association with plasma sCR1 changes.

C1s in the C1 complex cleaves C4 and C2 to initiate the classical complement pathway. Our interest in C1s was sparked by the recent report of a near-GWS association between rs3919533, a SNP located 5 kb upstream of *C1S,* and AD risk in European subjects [18]. No studies of C1s plasma levels in AD have been published, although C1s expression was decreased in CSF proteome of AD patients [61]. We found no difference in plasma C1s levels between AD patients and controls. Nevertheless, we investigated whether the GWAS-implicated SNP impacts plasma C1s levels and found that carriers of the minor allele at rs3919533 had significantly lower C1s levels. When plasma C1s levels were used as an intermediate phenotype in GWAS, we identified a cluster of highly significant SNPs located within *C1S*, including rs3919533, that associated with significantly decreased plasma C1s levels in both AD and control subjects. Taken together, the data suggest that variants in *C1S* may contribute to AD risk through changes in plasma C1s levels.

FH, an essential regulator of the alternative pathway, was previously reported to be decreased in plasma and CSF from AD patients, the latter specifically in amyloid positive cases [62, 63]. Consistent with these reports, we found a significant decrease in plasma FH in AD patients. Lower FH levels may impact regulation of the alternative pathway amplification loop. Although no variants in *CFH* have been reported to associate with AD risk in GWAS in Caucasian populations, there are numerous associations with other inflammatory diseases, notably AMD, a retinal neurodegenerative disease that has many similarities to AD [64, 65]. Two coding SNPs in *CFH*, rs1061170 (Y402H) and rs800292 (I62V) strongly impact risk of AMD. Although neither of these SNPs were significant in AD GWAS in Caucasians, both were strongly associated with AD risk and rate of atrophy in a Chinese cohort [22]. In a small case–control study in a Caucasian population, rs1061170 was associated with AD risk but only in individuals carrying the APOE*ε4* allele [23]. Although neither of these SNPs were significant in the FH endophenotype GWAS, the analysis identified a LD cluster of GWS SNPs in *CFH* and downstream, adjacent to *CFHR4*, that associated with changes in plasma FH levels. The lead variant, rs6664877, was associated with significantly increased plasma FH levels in both AD and control subjects. This intronic *CFH* SNP has not been previously described or related to any pathology, making it, together with the SNPs in strong LD, interesting candidates for further studies into roles of *CFH* variants in AD. A recent study of the genetic architecture of the human plasma proteome in healthy blood donors identified several associations between protein levels and complement genes; notably, variants in *CFH* significantly associated with 59 proteins [66].

## Conclusions

We demonstrate dysregulation of the complement system in AD plasma compared to controls. Clusterin and C1q were elevated, and sCR1 and FH decreased. C1q levels distinguished AD from controls with good predictive power particularly for LOAD (AUC 0.655). We show that SNPs in *CR1, C1S,* and *CFH*, some previously associated with AD, others novel, significantly influenced plasma concentrations of the respective proteins, suggesting a mechanism by which they impact disease risk. Although the changes observed are modest, we have shown before that even small changes in complement protein activities or levels can markedly impact risk of systemic diseases [67]. Limitations of the work relate to the nature of the cohort: 1. Assignation to AD or control groups was done by extensive cognitive testing in the cohort without recourse to imaging or CSF biomarker data; 2. The control and AD groups were not age-matched, indeed, controls were deliberately selected for advanced age with no evidence of cognitive impairment to exclude incipient cases, a clear advantage for genetic studies; 3. We split the AD cohort into early (onset before 65) and late (onset after 65), an arbitrary but useful distinction that can highlight early changes, but did not consider other potential stratifiers; 4. We did not include any individuals with mild cognitive impairment that could be followed over time, a future study could explore the time course of complement dysregulation in early disease to identify causative roles. Despite these limitations, our findings build a strong case for roles of genetically determined complement parameters in dictating risk of AD that may be useful in predicting AD and identifying novel routes to therapy.

## Supplementary Information

**Additional** **file** **1:** **Table** **S1.** Antibodies, proteins, and sample dilutions used in ELISA. All antibodies and standard proteins were produced in-house unless otherwise specified for each analyte. Comptech: Complement Technology, Inc. Abbreviations: mAb = monoclonal antibody, CV = coefficient of variation. **Table** **S2**. Pearson correlation scores between complement proteins and AT(N) biomarkers in control (A) and case samples (B). The top right triangle shows Pearson r correlation scores, while the bottom left triangle shows p values (italics). **Table** **S3**. Medians, Inter-Quartile Ranges (IQR) and Wilcoxon Rank Sum (Mann Whitney U) Test p-values for comparing distributions of plasma protein levels in early-onset Alzheimer’s disease (EOAD) vs. late-onset Alzheimer’s disease (LOAD) (* = p<0.05; ** = p<0.01; *** = p<0.001). **Table** **S4**. ROC-AUC statistics of stepAIC informed GLMs predicting disease status from plasma protein levels, adjusted for age and sex. Each stepAIC informed GLM model (Model), AUC values (AUC), Akaike Information Criterion (AIC), 95% confidence intervals (generated from 2000 bootstrap replicates) (CI), Z scores (Z), standard error (SE) and p-values (p-value) are given for each GLM and resultant ROC curve for predicting EOAD and LOAD status against controls. **Table** **S5.** Receiver-operator characteristic area under the curve (ROC-AUC) statistics for three major confounders (age, sex and APOE status), plus each protein biomarker, in generalised linear models (GLMs) to predict AD status and results of DeLong tests, comparing GLMs containing the three confounders and GLMs containing the three confounders plus each protein biomarker. Results are for Controls vs EOAD, Controls vs LOAD and EOAD vs LOAD, and each column contains the GLMs (Model), AUC of the resultant ROC curves, 95% confidence intervals (generated from 2000 bootstrap replicates) for the ROC-AUC (CI), Z-scores for the ROC-AUC (Z), standard error for the ROC-AUC (SE), p-values for the ROC-AUC (p-value) and confidence intervals, Z-scores and p-values from the results of the DeLong test (CI Delong, Z Delong, p-value Delong). **Table** **S6**. ROC-AUC statistics of GLMs predicting disease status from plasma protein levels, unadjusted for age and sex. For each protein, AUC values (AUC), 95% confidence intervals (from 2000 bootstrap replicates) (CI), Z scores (Z), standard error (SE) and p-values (p-value) are given for predicting EOAD and LOAD status vs controls, and EOAD vs LOAD. **Table** **S7**. ROC-AUC statistics of GLMs predicting disease status from plasma protein levels, adjusted for age and sex. For each protein, AUC values (AUC), 95% confidence intervals (from 2000 bootstrap replicates) (CI), Z scores (Z), standard error (SE) and p-values (p-value) are given for predicting EOAD and LOAD status vs controls, and EOAD vs LOAD. **Table** **S8.** Results of Generalised Linear Models (GLMs) estimating protein levels from Age, Sex and an interaction term between phenotype (Control vs EOAD, Control vs LOAD, Control vs AD) and certain genotypes (SNP rsIDs). Estimate values for each element (Estimate), the standard error (Std Error), the t-value statistic (t-value), significance thresholds (Pr(>|t|)) and formula used to create each GLM. **Figure** **S1**. Forest plot of regression coefficients (estimate values) of the generalised linear models (A = early-onset Alzheimer’s disease (EOAD), B = late-onset Alzheimer’s disease (LOAD)), showing the direction and strength of effect for ATN biomarkers and complement proteins measured for each AD group vs. controls. Proteins with negative coefficients are shown in green and those with a positive coefficient are shown in red. Proteins shown in grey were not statistically significant at the Bonferroni corrected p-value significance thresholds (* = p<0.005; ** = p<0.001; *** = p<0.0001). Error bars indicate the standard error for each regression coefficient. **Figure** **S2**. Density plots showing distributions of plasma protein levels (each protein labelled below the X-axes) for each of the groups (red = controls, green = early-onset Alzheimer’s disease (EOAD), blue = late-onset Alzheimer’s disease (LOAD)). **Figure** **S3**. Scatter plots showing distributions of plasma protein levels on the Y axes (each protein labelled on the Y-axes) vs age at inclusion on the X-axes for each of the groups (red = controls, green = early-onset Alzheimer’s disease (EOAD), blue = late-onset Alzheimer’s disease (LOAD)).

## Data Availability

Information on the data underpinning the results presented here, including how to access them, can be found in the Cardiff University data catalogue at [10.17035/d.2023.0272861462].

## Abbreviations

AD: Alzheimer’s disease
AUC: Area under curve
CSF: Cerebrospinal fluid
EOAD: Early onset AD
FH: Factor H
GFAP: Glial fibrillary acidic protein
GWAS: Genome-wide association study
GWS: Genome-wide significance
LOAD: Late onset AD
NfL: Neurofilament light
pTau: Phosphorylated tau
ROC: Receiver operating characteristic
sCR1: Soluble complement receptor 1
AMD: Age-related macular degeneration

## References

[1] Wittenberg R, Knapp M, Karagiannidou M, Dickson J, Schott JM (2019). Economic impacts of introducing diagnostics for mild cognitive impairment Alzheimer's disease patients. Alzheimers Dement (N Y).

[2] Hardy J, Selkoe DJ (2002). The amyloid hypothesis of Alzheimer's disease: progress and problems on the road to therapeutics. Science.

[3] Zolochevska O, Bjorklund N, Woltjer R, Wiktorowicz JE, Taglialatela G (2018). Postsynaptic proteome of non-demented individuals with Alzheimer's disease neuropathology. J Alzheimers Dis.

[4] Jack CR, Bennett DA, Blennow K, Carrillo MC, Dunn B, Haeberlein SB (2018). NIA-AA research framework: toward a biological definition of Alzheimer's disease. Alzheimers Dement.

[5] Alcolea D, Delaby C, Munoz L, Torres S, Estelles T, Zhu N (2021). Use of plasma biomarkers for AT(N) classification of neurodegenerative dementias. J Neurol Neurosurg Psychiatry.

[6] Palmqvist S, Tideman P, Cullen N, Zetterberg H, Blennow K, Dage JL (2021). Prediction of future Alzheimer's disease dementia using plasma phospho-tau combined with other accessible measures. Nat Med.

[7] Flores-Cordero JA, Perez-Perez A, Jimenez-Cortegana C, Alba G, Flores-Barragan A, Sanchez-Margalet V (2022). Obesity as a risk factor for dementia and Alzheimer's disease: the role of leptin. Int J Mol Sci.

[8] Athanasaki A, Melanis K, Tsantzali I, Stefanou MI, Ntymenou S, Paraskevas SG (2022). Type 2 diabetes mellitus as a risk factor for Alzheimer's disease: review and meta-analysis. Biomedicines.

[9] Zhang MN, Shi YD, Jiang HY (2022). The risk of dementia in patients with inflammatory bowel disease: a systematic review and meta-analysis. Int J Colorectal Dis.

[10] Khemka VK, Ganguly A, Bagchi D, Ghosh A, Bir A, Biswas A, Chattopadhyay S, Chakrabarti S (2014). Raised serum proinflammatory cytokines in Alzheimer's disease with depression. Aging Dis.

[11] McGeer PL, Akiyama H, Itagaki S, McGeer EG (1989). Activation of the classical complement pathway in brain-tissue of Alzheimer patients. Neurosci Lett.

[12] Zanjani H, Finch CE, Kemper C, Atkinson J, McKeel D, Morris JC (2005). Complement activation in very early Alzheimer disease. Alzheimer Dis Assoc Disord.

[13] Jiang HX, Burdick D, Glabe CG, Cotman CW, Tenner AJ (1994). Beta-amyloid activates complement by binding to a specific region of the collagen-like domain of the c1q a-chain. J Immunol.

[14] Geraghty NJ, Satapathy S, Kelly M, Cheng F, Lee A, Wilson MR (2021). Expanding the family of extracellular chaperones: identification of human plasma proteins with chaperone activity. Protein Sci.

[15] Deming Y, Xia J, Cai YF, Lord J, Holmans P, Bertelsen S (2016). A potential endophenotype for Alzheimer's disease: cerebrospinal fluid clusterin. Neurobiol Aging.

[16] Mullan GM, McEneny J, Fuchs M, McMaster C, Todd S, McGuinness B (2013). Plasma clusterin levels and the rs11136000 genotype in individuals with mild cognitive impairment and Alzheimer's disease. Curr Alzheimer Res.

[17] Sims R, Hill M, Williams J (2020). The multiplex model of the genetics of Alzheimer's disease. Nat Neurosci.

[18] Bellenguez C, Kucukali F, Jansen IE, Kleineidam L, Moreno-Grau S, Amin N (2022). New insights into the genetic etiology of Alzheimer's disease and related dementias. Nat Genet.

[19] Lambert JC, Heath S, Even G, Campion D, Sleegers K, Hiltunen M (2009). Genome-wide association study identifies variants at CLU and CR1 associated with Alzheimer's disease. Nat Genet.

[20] Thambisetty M, Beason-Held LL, An Y, Kraut M, Nalls M, Hernandez DG (2013). Alzheimer risk variant CLU and brain function during aging. Biol Psychiatry.

[21] Mahmoudi R, Kisserli A, Novella JL, Donvito B, Drame M, Reveil B (2015). Alzheimer's disease is associated with low density of the long CR1 isoform. Neurobiol Aging.

[22] Zhang DF, Li J, Wu H, Cui Y, Bi R, Zhou HJ (2016). CFH variants affect structural and functional brain changes and genetic risk of Alzheimer's disease. Neuropsychopharmacol.

[23] Zetterberg M, Landgren S, Andersson ME, Palmér MS, Gustafson DR, Skoog I (2008). Association of complement factor H Y402H gene polymorphism with Alzheimer's disease. Am J Med Genet B Neuropsychiatr Genet.

[24] Kim S, Swaminathan S, Shen L, Risacher SL, Nho K, Foroud T (2011). Genome-wide association study of CSF biomarkers A beta(1–42), t-tau, and p-tau(181p) in the ADNI cohort. Neurology.

[25] Kauwe JSK, Cruchaga C, Mayo K, Fenoglio C, Bertelsen S, Nowotny P (2008). Variation in MAPT is associated with cerebrospinal fluid tau levels in the presence of amyloid-beta deposition. Proc Nat Acad Sci USA.

[26] Shen L, Thompson PM, Potkin SG, Bertram L, Farrer LA, Foroud TM (2014). Genetic analysis of quantitative phenotypes in AD and MCI: imaging, cognition and biomarkers. Brain Imaging Behav.

[27] Cruchaga C, Kauwe JSK, Harari O, Jin SC, Cai YF, Karch CM (2013). GWAS of cerebrospinal fluid tau levels identifies risk variants for Alzheimer's disease. Neuron.

[28] Han MR, Schellenberg GD, Wang LS (2010). Alzheimer's Disease Neuroimaging Initiative. Genome-wide association reveals genetic effects on human Aβ42 and τ protein levels in cerebrospinal fluids: a case control study. BMC Neurol.

[29] Potkin SG, Turner JA, Guffanti G, Lakatos A, Fallon JH, Nguyen DD (2009). A genome-wide association study of schizophrenia using brain activation as a quantitative phenotype. Schizophr Bull.

[30] Cruchaga C, Ebbert MW, Kauwe JK (2014). Genetic discoveries in AD using CSF amyloid and tau. Curr Genet Med Rep.

[31] Holmes C, Cairns N, Lantos P, Mann A (1999). Validity of current clinical criteria for Alzheimer's disease, vascular dementia and dementia with Lewy bodies. Br J Psychiatry.

[32] Abraham R, Moskvina V, Sims R, Hollingworth P, Morgan A, Georgieva L (2008). A genome-wide association study for late-onset Alzheimer's disease using DNA pooling. Bmc Med Genom.

[33] Leonenko G, Baker E, Stevenson-Hoare J, Sierksma A, Fiers M, Williams J (2021). Identifying individuals with high risk of Alzheimer's disease using polygenic risk scores. Nat Commun.

[34] Stevenson-Hoare J, Heslegrave A, Leonenko G, Fathalla D, Bellou E, Luckcuck L (2022). Plasma biomarkers and genetics in the diagnosis and prediction of Alzheimer’s disease. Brain.

[35] Harold D, Abraham R, Hollingworth P, Sims R, Gerrish A, Hamshere ML (2009). Genome-wide association study identifies variants at CLU and PICALM associated with Alzheimer's disease. Nat Genet.

[36] Das S, Forer L, Schonherr S, Sidore C, Locke AE, Kwong A (2016). Next-generation genotype imputation service and methods. Nat Genet.

[37] The 1000 Genomes Project Consortium. A global reference for human genetic variation. Nature. 2015;526(7571):68–74. 10.1038/nature15393 PMC4750478 26432245

[38] Chang CC, Chow CC, Tellier L, Vattikuti S, Purcell SM, Lee JJ (2015). Second-generation PLINK: rising to the challenge of larger and richer datasets. Gigascience.

[39] Boughton AP, Welch RP, Flickinger M, VandeHaar P, Taliun D, Abecasis GR, Boehnke M (2021). LocusZoom.js: interactive and embeddable visualization of genetic association study results. Bioinformatics.

[40] Yasojima K, Schwab C, McGeer EG, McGeer PL (1999). Up-regulated production and activation of the complement system in Alzheimer's disease brain. Am J Pathol.

[41] Webster SD, Yang AJ, Margol L, Garzon-Rodriguez W, Glabe CG, Tenner AJ (2000). Complement component C1q modulates the phagocytosis of A beta by microglia. Exp Neurol.

[42] Hasegawa N, Fujie S, Horii N, Uchida M, Toyama Y, Inoue K (2019). Aging-induced elevation in circulating complement C1q level is associated with arterial stiffness. Exp Gerontol.

[43] Beeg M, Stravalaci M, Romeo M, Carra AD, Cagnotto A, Rossi A (2016). Clusterin binds to A(1–42) oligomers with high affinity and interferes with peptide aggregation by inhibiting primary and secondary nucleation. J Biol Chem.

[44] Bell RD, Sagare AP, Friedman AE, Bedi GS, Holtzman DM, Deane R (2007). Transport pathways for clearance of human Alzheimer's amyloid beta-peptide and apolipoproteins E and J in the mouse central nervous system. J Cereb Blood Flow Metab.

[45] Wojtas AM, Kang SS, Olley BM, Gatherer M, Shinohara M, Lozano PA (2017). Loss of clusterin shifts amyloid deposition to the cerebrovasculature via disruption of perivascular drainage pathways. Proc Nat Acad Sci USA.

[46] Schurmann B, Wiese B, Bickel H, Weyerer S, Riedel-Heller SG, Pentzek M (2011). Association of the Alzheimer's disease clusterin risk allele with plasma clusterin concentration. J Alzheimers Dis.

[47] Hsu JL, Lee WJ, Liao YC, Wang SJ, Fuh JL (2017). The clinical significance of plasma clusterin and A beta in the longitudinal follow-up of patients with Alzheimer's disease. Alzheimers Res Ther.

[48] Narayan P, Orte A, Clarke RW, Bolognesi B, Hook S, Ganzinger KA (2011). The extracellular chaperone clusterin sequesters oligomeric forms of the amyloid-beta(1–40) peptide. Nat Struct Mol Biol.

[49] Yerbury JJ, Poon S, Meehan S, Thompson B, Kumita JR, Dobson CM, Wilson MR (2007). The extracellular chaperone clusterin influences amyloid formation and toxicity by interacting with prefibrillar structures. FASEB.

[50] Shayo M, McLay RN, Kastin AJ, Banks WA (1997). The putative blood–brain barrier transporter for the beta-amyloid binding protein apolipoprotein J is saturated at physiological concentrations. Life Sci.

[51] Thambisetty M, Simmons A, Velayudhan L, Hye A, Campbell J, Zhang Y (2010). Association of plasma clusterin concentration with severity, pathology, and progression in Alzheimer disease. Arch Gen Psychiatry.

[52] Jackson RJ, Rose J, Tulloch J, Henstridge C, Smith C, Spires-Jones TL (2019). Clusterin accumulates in synapses in Alzheimer's disease and is increased in apolipoprotein E4 carriers. Brain Commun..

[53] Song F, Poljak A, Crawford J, Kochan NA, Wen W, Cameron B (2012). Plasma apolipoprotein levels are associated with cognitive status and decline in a community cohort of older individuals. PLoS ONE.

[54] Montanola A, de Retana SF, Lopez-Rueda A, Merino-Zamorano C, Penalba A, Fernandez-Alvarez P (2016). ApoA1, ApoJ and ApoE plasma levels and genotype frequencies in cerebral amyloid angiopathy. Neuromolecular Med.

[55] Daskoulidou N, Shaw B, Torvell M, Watkins L, Cope EL, Carpanini SM (2023). Complement receptor 1 is expressed on brain cells and in the human brain. Glia.

[56] Morgan AR, Touchard S, Leckey C, O'Hagan C, Nevado-Holgado AJ, Barkhof F (2019). Inflammatory biomarkers in Alzheimer's disease plasma. Alzheimers Dement.

[57] Mahmoudi R, Feldman S, Kisserli A, Duret V, Tabary T, Bertholon LA (2018). Inherited and acquired decrease in complement receptor 1 (CR1) density on red blood cells associated with high levels of soluble CR1 in Alzheimer's disease. Int J Mol Sci.

[58] Chibnik LB, Shulman JM, Leurgans SE, Schneider JA, Wilson RS, Tran D (2011). CR1 is associated with amyloid plaque burden and age-related cognitive decline. Ann Neurol.

[59] Fonseca MI, Chu SH, Pierce AL, Brubaker WD, Hauhart RE, Mastroeni D (2016). Analysis of the putative role of CR1 in Alzheimer's disease: genetic association, expression and function. PLoS ONE.

[60] Zhu XC, Wang HF, Jiang T, Lu H, Tan MS, Tan CC (2017). Effect of CR1 genetic variants on cerebrospinal fluid and neuroimaging biomarkers in healthy, mild cognitive impairment and Alzheimer's disease cohorts. Mol Neurobiol.

[61] Khoonsari PE, Haggmark A, Lonnberg M, Mikus M, Kilander L, Lannfelt L (2016). Analysis of the cerebrospinal fluid proteome in Alzheimer's disease. PLoS ONE.

[62] Lu G, Liu WH, Huang XY, Zhao YX (2020). Complement factor H levels are decreased and correlated with serum C-reactive protein in late-onset Alzheimer's disease. Arq Neuro-Psiquiatr.

[63] Brosseron F, Kolbe CC, Santarelli F, Carvalho S, Antonell A, Castro-Gomez S (2020). Multicenter Alzheimer's and Parkinson's disease immune biomarker verification study. Alzheimers Dement.

[64] Ashok A, Singh N, Chaudhary S, Bellamkonda V, Kritikos AE, Wise AS (2020). Retinal degeneration and Alzheimer's disease: an evolving link. Int J Mol Sci.

[65] Toomey CB, Johnson LV, Bowes RC (2018). Complement factor H in AMD: bridging genetic associations and pathobiology. Prog Retin Eye Res.

[66] Sun BB, Maranville JC, Peters JE, Stacey D, Staley JR, Blackshaw J (2018). Genomic atlas of the human plasma proteome. Nature.

[67] Harris CL, Heurich M, de Cordoba SR, Morgan BP (2012). The complotype: dictating risk for inflammation and infection. Trends Immunol.

