# Supplementary material for: Alzheimer’s disease-associated complement gene variants influence plasma complement protein levels
Source: J Neuroinflammation. 2023 Jul 21;20:169. doi: 10.1186/s12974-023-02850-6 (PMC10362776; doi:10.1186/s12974-023-02850-6)
Supplement: Supplementary file 1 — Additional file 1: Table S1. Antibodies, proteins, and sample dilutions used in ELISA. All antibodies and standard proteins were produced in-house unless otherwise specified for each analyte. Comptech: Complement Technology, Inc. Abbreviations: mAb = monoclonal antibody, CV = coefficient of variation. Table S2. Pearson correlation scores between complement proteins and AT(N) biomarkers in control (A) and case samples (B). The top right triangle shows Pearson r correlation scores, while the bottom left triangle shows p values (italics). Table S3. Medians, Inter-Quartile Ranges (IQR) and Wilcoxon Rank Sum (Mann Whitney U) Test p-values for comparing distributions of plasma protein levels in early-onset Alzheimer’s disease (EOAD) vs. late-onset Alzheimer’s disease (LOAD) (* = p<0.05; ** = p<0.01; *** = p<0.001). Table S4. ROC-AUC statistics of stepAIC informed GLMs predicting disease status from plasma protein levels, adjusted for age and sex. Each stepAIC informed GLM model (Model), AUC values (AUC), Akaike Information Criterion (AIC), 95% confidence intervals (generated from 2000 bootstrap replicates) (CI), Z scores (Z), standard error (SE) and p-values (p-value) are given for each GLM and resultant ROC curve for predicting EOAD and LOAD status against controls. Table S5. Receiver-operator characteristic area under the curve (ROC-AUC) statistics for three major confounders (age, sex and APOE status), plus each protein biomarker, in generalised linear models (GLMs) to predict AD status and results of DeLong tests, comparing GLMs containing the three confounders and GLMs containing the three confounders plus each protein biomarker. Results are for Controls vs EOAD, Controls vs LOAD and EOAD vs LOAD, and each column contains the GLMs (Model), AUC of the resultant ROC curves, 95% confidence intervals (generated from 2000 bootstrap replicates) for the ROC-AUC (CI), Z-scores for the ROC-AUC (Z), standard error for the ROC-AUC (SE), p-values for the ROC-AUC (p-value) and co [file 12974_2023_2850_MOESM1_ESM.docx]

| **Biomarker** | **Capture antibody** | **Detection antibody** | **Standard protein** | **Plasma dilution** | **Lower/upper limit of detection (ng/ml)** | **Intra-/Inter-assay CV (%)** |
| --- | --- | --- | --- | --- | --- | --- |
| C1q | mAb 9H10 (2 µg/ml) | Rabbit α-C1q (1 µg/ml) | C1q | 1:16000 | 16-1000 | 5.6/11.5 |
| CR1 | Rabbit α-CR1 (2.5 µg/ml) | mAb MBI35 (1 µg/ml) | CR1 | 1:5 | 2-100 | 3.7/10.3 |
| FH | mAb OX-24 (2 µg/ml) | mAb 35H9 (2 µg/ml) | Factor H | 1:2000 | 1.5-1500 | 3.9/13.4 |
| Clusterin | mAb 2D5 (5 µg/ml) | mAb 4C7-HRP (2 µg/ml) | Clusterin | 1:2000 | 8-2000 | 4/10.5 |
| C1s | Goat α-C1s (Quidel) (20 µg/ml) | mAb M81 (Hycult) (2 µg/ml) | C1s (Comptech) | 1:200 | 8-2000 | 3/14 |

**Table S1. Antibodies, proteins, and sample dilutions used in ELISA.** All antibodies and standard proteins were produced in-house unless otherwise specified for each analyte. Comptech: Complement Technology, Inc. Abbreviations: mAb = monoclonal antibody, CV = coefficient of variation.

**Table S2.** **Pearson correlation scores between complement proteins and AT(N) biomarkers in control (A) and case samples (B).** The top right triangle shows Pearson r correlation scores, while the bottom left triangle shows p values (italics).

1. Controls

| **CONTROL** | **Clusterin** | **sCR1** | **FH** | **C1q** | **C1s** | **Aβ40** | **Aβ42** | **GFAP** | **NfL** | **pTau181** |
| --- | --- | --- | --- | --- | --- | --- | --- | --- | --- | --- |
| **Clusterin** |  | 0.047 | 0.055 | 0.047 | -0.010 | 0.042 | 0.047 | 0.076 | 0.138 | -0.032 |
| **sCR1** | *0.293* |  | 0.083 | 0.109 | 0.076 | 0.020 | 0.043 | 0.176 | 0.115 | 0.060 |
| **FH** | *0.222* | *0.063* |  | 0.402 | 0.215 | 0.155 | 0.055 | 0.012 | 0.054 | -0.062 |
| **C1q** | *0.290* | *0.015* | *0.000* |  | 0.235 | 0.155 | 0.081 | 0.023 | 0.033 | -0.024 |
| **C1s** | *0.827* | *0.090* | *0.000* | *0.000* |  | -0.011 | -0.038 | -0.001 | 0.001 | -0.058 |
| **Aβ40** | *0.364* | *0.664* | *0.001* | *0.001* | *0.816* |  | 0.705 | 0.464 | 0.532 | 0.200 |
| **Aβ42** | *0.305* | *0.355* | *0.237* | *0.078* | *0.405* | *0.000* |  | 0.288 | 0.399 | 0.050 |
| **GFAP** | *0.093* | *0.000* | *0.798* | *0.607* | *0.974* | *0.000* | *0.000* |  | 0.532 | 0.316 |
| **NfL** | *0.003* | *0.013* | *0.247* | *0.482* | *0.982* | *0.000* | *0.000* | *0.000* |  | 0.307 |
| **pTau181** | *0.491* | *0.204* | *0.187* | *0.604* | *0.212* | *0.000* | *0.297* | *0.000* | *0.000* |  |

(B) Cases

| **AD** | **Clusterin** | **sCR1** | **FH** | **C1q** | **C1s** | **Aβ40** | **Aβ42** | **GFAP** | **NfL** | **pTau181** |
| --- | --- | --- | --- | --- | --- | --- | --- | --- | --- | --- |
| **Clusterin** |  | -0.027 | 0.014 | 0.052 | 0.049 | -0.118 | -0.093 | -0.016 | -0.056 | -0.037 |
| **sCR1** | *0.306* |  | 0.102 | 0.110 | 0.128 | 0.007 | 0.000 | 0.027 | 0.068 | 0.047 |
| **FH** | *0.589* | *0.000* |  | 0.262 | 0.238 | 0.052 | 0.030 | -0.032 | 0.019 | -0.061 |
| **C1q** | *0.053* | *0.000* | *0.000* |  | 0.212 | -0.093 | -0.093 | -0.072 | -0.042 | -0.038 |
| **C1s** | *0.070* | *0.000* | *0.000* | *0.000* |  | 0.051 | 0.060 | 0.006 | 0.006 | 0.010 |
| **Aβ40** | *0.000* | *0.807* | *0.056* | *0.001* | *0.059* |  | 0.776 | 0.258 | 0.317 | 0.091 |
| **Aβ42** | *0.001* | *0.993* | *0.273* | *0.001* | *0.028* | *0.000* |  | 0.160 | 0.243 | 0.000 |
| **GFAP** | *0.560* | *0.324* | *0.243* | *0.008* | *0.826* | *0.000* | *0.000* |  | 0.552 | 0.550 |
| **NfL** | *0.043* | *0.013* | *0.502* | *0.129* | *0.823* | *0.000* | *0.000* | *0.000* |  | 0.403 |
| **pTau181** | *0.179* | *0.085* | *0.025* | *0.165* | *0.704* | *0.001* | *0.993* | *0.000* | *0.000* |  |

**Table S3.** **Medians, Inter-Quartile Ranges (IQR) and Wilcoxon Rank Sum (Mann Whitney U) Test p-values** for comparing distributions of plasma protein levels in early-onset Alzheimer’s disease (EOAD) vs. late-onset Alzheimer’s disease (LOAD) (* = p<0.05; ** = p<0.01; *** = p<0.001).

| Protein | Control Median | Control IQR | EOAD Median | EOAD IQR | LOAD Median | LOAD IQR | p-value |
| --- | --- | --- | --- | --- | --- | --- | --- |
| Aβ40 (pg/ml) *** | 138.969 | 49.741 | 83.4818 | 35.412 | 95.909 | 95.909 | 4.72e-18 |
| Aβ42 (pg/ml) *** | 7.433 | 2.766 | 4.493 | 1.7867 | 5.234 | 5.234 | 2.25e-15 |
| Aβ40/42 | 0.0539 | 0.0127 | 0.0539 | 0.0127 | 0.0528 | 0.0528 | 3.93e-01 |
| pTau181 (pg/ml) * | 2.743 | 1.504 | 3.862 | 2.667 | 4.0866 | 4.0865 | 2.50e-02 |
| NfL (pg/ml) *** | 30.641 | 14.85 | 25.403 | 15.879 | 33.603 | 33.603 | 5.83e-24 |
| GFAP (pg/ml) *** | 177.483 | 97.382 | 186.035 | 123.958 | 228.357 | 228.357 | 1.22e-12 |
| Clusterin (µgml) ** | 193.014 | 85.0321 | 219.815 | 82.709 | 207.591 | 207.591 | 1.14e-04 |
| CR1 (ng/ml) * | 15.031 | 6.895 | 14.656 | 5.916 | 13.885 | 13.885 | 2.44e-02 |
| C1q (µgml) | 101.347 | 50.192 | 143.733 | 62.945 | 139.804 | 139.804 | 8.31e-02 |
| C1s (µgml) | 26.704 | 10.495 | 27.19 | 10.377 | 26.876 | 26.876 | 3.37e-01 |
| Factor H (µg/ml) | 365.869 | 180.468 | 331.605 | 148.949 | 332.13936 | 332.139 | 8.72e-01 |

**Table S4. ROC-AUC statistics of stepAIC informed GLMs predicting disease status from plasma protein levels, adjusted for age and sex.** Each stepAIC informed GLM model (**Model**), AUC values (**AUC**), Akaike Information Criterion (**AIC**), 95% confidence intervals (generated from 2000 bootstrap replicates) (**CI**), Z scores (**Z**), standard error (**SE**) and p-values (**p-value**) are given for each GLM and resultant ROC curve for predicting **EOAD** and **LOAD** status against controls.

**Early Onset Alzheimer’s Disease (EOAD)**

| Model | AUC | AIC | CI | Z | SE | p-value |
| --- | --- | --- | --- | --- | --- | --- |
| Aß40 + Aß40/42 + GFAP + C1q | 0.681 | 1064.17 | 0.629-0.737 | -4.758 | 0.079 | 1.95e-06 |
| Aß40 + Aß40/42 + NfL + GFAP + C1q | 0.686 | 1064.34 | 0.635-0.74 | -4.908 | 0.08 | 9.19e-07 |
| Aß40 + Aß40/42 + NfL + GFAP + Clusterin + C1q | 0.694 | 1065.42 | 0.64-0.746 | -4.814 | 0.081 | 1.48e-06 |
| Aß40 + Aß40/42 + NfL + GFAP + Clusterin + C1q + Factor H | 0.700 | 1066.57 | 0.65-0.753 | -4.638 | 0.081 | 3.52e-06 |

**Late Onset Alzheimer’s Disease (LOAD)**

| Model | AUC | AIC | CI | Z | SE | p-value |
| --- | --- | --- | --- | --- | --- | --- |
| Aß40 + Aß40/42 + pTau181 + NfL + GFAP + CR1 + C1q + Factor H | 0.824 | 644.41 | 0.771-0.874 | -7.024 | 0.121 | 2.16e-12 |
| Aß40 + Aß40/42 + pTau181 + NfL + GFAP + Clusterin + CR1 + C1q + Factor H | 0.825 | 645.21 | 0.773-0.873 | -6.995 | 0.121 | 2.65e-12 |
| Aß40 + Aß42 + Aß40/42 + pTau181 + NfL + GFAP + Clusterin + CR1 + C1q + Factor H | 0.825 | 647.18 | 0.771-0.873 | -2.162 | 0.363 | 3.06e-02 |
| Aß40 + Aß42 + Aß40/42 + pTau181 + NfL + GFAP + Clusterin + CR1 + C1q + C1s + Factor H | 0.825 | 649.17 | 0.774-0.874 | -2.16 | 0.363 | 3.08e-02 |

**Table S5.** **Receiver-operator characteristic area under the curve (ROC-AUC) statistics** for three major confounders (age, sex and APOE status), plus each protein biomarker, in generalised linear models (GLMs) to predict AD status and **results of DeLong tests**, comparing GLMs containing the three confounders and GLMs containing the three confounders plus each protein biomarker. Results are for **Controls vs EOAD**, **Controls vs LOAD** and **EOAD vs LOAD**, and each column contains the GLMs (**Model**), **AUC** of the resultant ROC curves, 95% confidence intervals (generated from 2000 bootstrap replicates) for the ROC-AUC (**CI**), Z-scores for the ROC-AUC (**Z**), standard error for the ROC-AUC (**SE**), p-values for the ROC-AUC (p-value) and confidence intervals, Z-scores and p-values from the results of the DeLong test (**CI Delong**, **Z Delong**, **p-value Delong**).

**Early Onset Alzheimer’s Disease (EOAD)**

| Model | AUC | CI | Z | SE | p-value | CI  Delong | Z  Delong | p-value Delong |
| --- | --- | --- | --- | --- | --- | --- | --- | --- |
| Age  +Sex  +APOE | 0.974 | 0.951-0.99 | 10.481 | 2.834 | 1.05E-25 | NA | NA | NA |
| +Aβ40 | 0.977 | 0.956-0.992 | 9.389 | 2.934 | 6.03E-21 | 8.21e-04 | -1.56 | 1.19e-01 |
| +Aβ42 | 0.978 | 0.958-0.993 | 9.351 | 2.997 | 8.69E-21 | 2.34e-03 | -1.28 | 1.99e-01 |
| +Aβ40/42 | 0.974 | 0.951-0.99 | 10.514 | 2.887 | 7.46E-26 | 3.00e-03 | 0.127 | 8.99e-01 |
| +pTau181 | 0.979 | 0.957-0.994 | 10.252 | 3.084 | 1.16E-24 | -6.20e-04 | -2.26 | 2.37e-02 |
| +NfL | 0.979 | 0.959-0.993 | 10.159 | 3.152 | 3.01E-24 | -1.01e-04 | -2.43 | 1.53e-02 |
| +GFAP | 0.98 | 0.96-0.994 | 10.106 | 3.32 | 5.20E-24 | -8.54e-05 | -1.99 | 4.70e-02 |
| +Clusterin | 0.981 | 0.961-0.994 | 10.332 | 2.926 | 5.07E-25 | -1.51e-03 | -2.54 | 1.12e-02 |
| +CR1 | 0.974 | 0.951-0.99 | 10.477 | 2.836 | 1.10E-25 | 6.49e-05 | -1.63 | 1.04e-01 |
| +C1q | 0.983 | 0.964-0.995 | 9.986 | 2.954 | 1.76E-23 | -2.33e-03 | -2.67 | 7.49e-03 |
| +C1s | 0.974 | 0.951-0.99 | 10.44 | 2.838 | 1.63E-25 | 1.21e-03 | -0.0775 | 9.38e-01 |
| +FactorH | 0.975 | 0.953-0.991 | 10.442 | 2.865 | 1.59E-25 | 8.07e-04 | -1.24 | 2.16e-01 |

**Late Onset Alzheimer’s Disease (LOAD)**

| Model | AUC | CI | Z | SE | p-value | CI  Delong | Z Delong | p-value Delong |
| --- | --- | --- | --- | --- | --- | --- | --- | --- |
| Age  +Sex  +APOE | 0.819 | 0.762-0.871 | 6.457 | 1.727 | 1.07e-10 | NA | NA | NA |
| +Aβ40 | 0.838 | 0.783-0.886 | 4.454 | 1.797 | 8.44e-06 | 1.1.7e-03 | -1.84 | 6.51e-02 |
| +Aβ42 | 0.842 | 0.789-0.89 | 4.695 | 1.802 | 2.67e-06 | 3.71e-03 | -1.69 | 9.18e-02 |
| +Aβ40/42 | 0.819 | 0.762-0.872 | 6.581 | 1.758 | 4.68e-11 | 7.12e-03 | -0.0358 | 9.71e-01 |
| +pTau181 | 0.854 | 0.803-0.903 | 7.124 | 1.853 | 1.05e-12 | -7.59e-03 | -2.49 | 1.26e-02 |
| +NfL | 0.863 | 0.813-0.908 | 7.552 | 1.939 | 4.29e-14 | -1.78e-02 | -3.29 | 1.02e-03 |
| +GFAP | 0.837 | 0.784-0.886 | 7.944 | 1.984 | 1.97e-15 | 9.03e-03 | -1.31 | 1.91e-01 |
| +Clusterin | 0.82 | 0.764-0.872 | 6.297 | 1.738 | 3.03e-10 | 9.98e-03 | -0.133 | 8.94e-01 |
| +CR1 | 0.822 | 0.765-0.875 | 6.481 | 1.731 | 9.14e-10 | 2.14e-03 | -1.21 | 2.27e-01 |
| +C1q | 0.849 | 0.797-0.894 | 6.007 | 1.781 | 1.89e-09 | -7.69e-03 | -2.63 | 8.58e-03 |
| +C1s | 0.821 | 0.764-0.873 | 6.481 | 1.727 | 9.12e-11 | 8.13e-04 | -1.35 | 1.78e-01 |
| +FactorH | 0.825 | 0.769-0.877 | 6.503 | 1.734 | 7.88e-11 | 3.13e-03 | -1.26 | 2.06e-01 |

**EOAD vs LOAD**

| Model | AUC | CI | Z | SE | p-value | CI  Delong | Z Delong | p-value Delong |
| --- | --- | --- | --- | --- | --- | --- | --- | --- |
| Age  +Sex  +APOE | 0.976 | 0.962-0.986 | -12.644 | 3.048 | 1.21e-36 | NA | NA | NA |
| +Aβ40 | 0.976 | 0.963-0.986 | -12.573 | 3.055 | 2.97e-36 | 1.59e-03 | -0.358 | 7.21e-01 |
| +Aβ42 | 0.975 | 0.961-0.986 | -12.581 | 3.059 | 2.67e-36 | 3.68e-03 | 0.608 | 5.43e-01 |
| +Aβ40/42 | 0.974 | 0.961-0.985 | -12.652 | 3.047 | 1.09e-36 | 2.36e-03 | 1.52 | 1.28e-01 |
| +pTau181 | 0.974 | 0.96-0.985 | -12.536 | 3.163 | 4.74e-36 | 4.74e-03 | 1.11 | 2.69e-01 |
| +NfL | 0.975 | 0.962-0.986 | -12.599 | 3.083 | 2.13e-36 | 1.45e-03 | 0.424 | 6.72e-02 |
| +GFAP | 0.974 | 0.961-0.985 | -12.324 | 3.241 | 6.69e-35 | 3.99e-03 | 1.28 | 1.99e-01 |
| +Clusterin | 0.976 | 0.963-0.986 | -12.566 | 3.109 | 3.24e-36 | 2.25e-03 | 0 | 1 |
| +CR1 | 0.976 | 0.963-0.986 | -12.639 | 3.048 | 1.28e-36 | 9.16e-04 | -0.293 | 7.69e-01 |
| +C1q | 0.975 | 0.962-0.986 | -12.601 | 3.053 | 2.08e-36 | 1.26e-03 | 0.87 | 3.84e-01 |
| +C1s | 0.975 | 0.962-0.986 | -12.647 | 3.081 | 1.16e-36 | 3.77e-03 | 0.405 | 6.85e-01 |
| +FactorH | 0.973 | 0.959-0.984 | -12.61 | 3.043 | 1.86e-36 | 4.87e-03 | 2.53 | 1.14e-02 |

**Table S6. ROC-AUC statistics of GLMs predicting disease status from plasma protein levels, unadjusted for age and sex.** For each protein, AUC values (**AUC**), 95% confidence intervals (from 2000 bootstrap replicates) (**CI**), Z scores (**Z**), standard error (**SE**) and p-values (**p-value**) are given for predicting **EOAD** and **LOAD** status vs controls, and **EOAD vs LOAD**.

**Early Onset Alzheimer’s Disease (EOAD)**

| Protein | AUC | CI | Z | SE | p-value |
| --- | --- | --- | --- | --- | --- |
| Aβ40 | 0.875 | 0.835-0.913 | -14.348 | 0.125 | 1.09e-46 |
| Aβ42 | 0.873 | 0.832-0.91 | -14.279 | 0.132 | 2.94e-46 |
| Aβ40/42 | 0.52 | 0.458-0.58 | -1.329 | 0.079 | 1.84e-01 |
| pTau181 | 0.668 | 0.613-0.725 | 6.518 | 0.086 | 7.14e-11 |
| NfL | 0.593 | 0.533-0.65 | -6.088 | 0.077 | 1.14e-09 |
| GFAP | 0.508 | 0.448-0.565 | 0.575 | 0.074 | 5.65e-01 |
| Clusterin | 0.638 | 0.578-0.692 | 5.055 | 0.082 | 4.30e-07 |
| CR1 | 0.557 | 0.495-0.622 | -3.185 | 0.07 | 1.45e-03 |
| C1q | 0.78 | 0.729-0.831 | 10.134 | 0.107 | 3.89e-24 |
| C1s | 0.512 | 0.45-0.573 | 1.407 | 0.073 | 1.59e-01 |
| Factor H | 0.564 | 0.503-0.627 | -2.634 | 0.07 | 8.43e-03 |

**Late Onset Alzheimer’s Disease (LOAD)**

| Protein | AUC | CI | Z | SE | p-value |
| --- | --- | --- | --- | --- | --- |
| Aβ40 | 0.764 | 0.703-0.82 | -9.861 | 0.112 | 6.17e-23 |
| Aβ42 | 0.772 | 0.709-0.829 | -10.315 | 0.123 | 6.01e-25 |
| Aβ40/42 | 0.541 | 0.471-0.612 | -0.847 | 0.081 | 3.97e-01 |
| pTau181 | 0.71 | 0.644-0.772 | 7.4 | 0.103 | 1.36e-13 |
| NfL | 0.571 | 0.503-0.639 | 1.928 | 0.084 | 5.39e-02 |
| GFAP | 0.637 | 0.57-0.703 | 5.883 | 0.093 | 4.02e-09 |
| Clusterin | 0.546 | 0.474-0.62 | 2.363 | 0.083 | 1.81e-02 |
| CR1 | 0.595 | 0.526-0.663 | -2.491 | 0.1 | 1.27e-02 |
| C1q | 0.738 | 0.675-0.797 | 8.264 | 0.103 | 1.41e-16 |
| C1s | 0.529 | 0.457-0.605 | -0.451 | 0.085 | 6.52e-01 |
| Factor H | 0.466 | 0.399-0.54 | -2.578 | 0.088 | 9.95e-03 |

**EOAD vs LOAD**

| Protein | AUC | CI | Z | SE | p-value |
| --- | --- | --- | --- | --- | --- |
| Aβ40 | 0.688 | 0.627-0.741 | 5.906 | 0.075 | 3.50e-09 |
| Aβ42 | 0.649 | 0.582-0.709 | 5.829 | 0.078 | 5.57e-09 |
| Aβ40/42 | 0.564 | 0.504-0.623 | 0.399 | 0.082 | 6.90e-01 |
| pTau181 | 0.556 | 0.499-0.616 | 1.745 | 0.071 | 8.10e-02 |
| NfL | 0.698 | 0.645-0.751 | 7.293 | 0.076 | 3.03e-13 |
| GFAP | 0.637 | 0.579-0.692 | 5.437 | 0.072 | 5.41e-08 |
| Clusterin | 0.559 | 0.498-0.62 | -2.929 | 0.074 | 3.40e-03 |
| CR1 | 0.539 | 0.48-0.601 | -0.866 | 0.074 | 3.87e-01 |
| C1q | 0.502 | 0.438-0.56 | -2.364 | 0.072 | 1.81e-02 |
| C1s | 0.486 | 0.423-0.549 | -1.089 | 0.074 | 2.76e-01 |
| Factor H | 0.563 | 0.5-0.623 | -1.21 | 0.073 | 2.26e-01 |

**Table S7. ROC-AUC statistics of GLMs predicting disease status from plasma protein levels, adjusted for age and sex.** For each protein, AUC values (**AUC**), 95% confidence intervals (from 2000 bootstrap replicates) (**CI**), Z scores (**Z**), standard error (**SE**) and p-values (**p-value**) are given for predicting **EOAD** and **LOAD** status vs controls, and **EOAD vs LOAD**.

**Early-Onset Alzheimer’s Disease (EOAD)**

| Protein | AUC | CI | Z | SE | p-value |
| --- | --- | --- | --- | --- | --- |
| Aβ40 | 0.616 | 0.557-0.679 | -3.565 | 0.073 | 3.64e-04 |
| Aβ42 | 0.611 | 0.553-0.675 | -4.918 | 0.074 | 8.74e-07 |
| Aβ40/42 | 0.491 | 0.432-0.547 | -2.662 | 0.073 | 7.76e-03 |
| pTau181 | 0.57 | 0.518-0.634 | 2.663 | 0.075 | 7.75e-03 |
| NfL | 0.576 | 0.515-0.639 | 2.093 | 0.075 | 3.64e-02 |
| GFAP | 0.581 | 0.522-0.639 | 3.12 | 0.076 | 1.81e-03 |
| Clusterin | 0.581 | 0.522-0.636 | 1.696 | 0.074 | 8.99e-02 |
| CR1 | 0.543 | 0.478-0.606 | -0.052 | 0.072 | 9.59e-01 |
| C1q | 0.601 | 0.543-0.664 | 2.945 | 0.077 | 3.23e-03 |
| C1s | 0.516 | 0.457-0.575 | 0.111 | 0.072 | 9.11e-01 |
| Factor H | 0.528 | 0.465-0.591 | -1.015 | 0.072 | 3.10e-01 |

**Late-Onset Alzheimer’s Disease (LOAD)**

| Protein | AUC | CI | Z | SE | p-value |
| --- | --- | --- | --- | --- | --- |
| Aβ40 | 0.657 | 0.589-0.722 | -5.373 | 0.09 | 7.74e-08 |
| Aβ42 | 0.665 | 0.599-0.733 | -6.343 | 0.093 | 2.25e-10 |
| Aβ40/42 | 0.561 | 0.492-0.63 | -1.381 | 0.084 | 1.67e-01 |
| pTau181 | 0.669 | 0.605-0.738 | 6.645 | 0.1 | 3.03e-11 |
| NfL | 0.674 | 0.609-0.734 | 5.492 | 0.091 | 3.97e-08 |
| GFAP | 0.67 | 0.607-0.734 | 7.2 | 0.102 | 6.02e-13 |
| Clusterin | 0.529 | 0.459-0.601 | 1.762 | 0.085 | 7.80e-02 |
| CR1 | 0.554 | 0.484-0.624 | -1.155 | 0.084 | 2.48e-01 |
| C1q | 0.655 | 0.587-0.718 | 5.676 | 0.092 | 1.38e-08 |
| C1s | 0.546 | 0.475-0.622 | 0.362 | 0.083 | 7.17e-01 |
| Factor H | 0.533 | 0.459-0.598 | -2.065 | 0.084 | 3.90e-02 |

**EOAD vs LOAD**

| Protein | AUC | CI | Z | SE | p-value |
| --- | --- | --- | --- | --- | --- |
| Aβ40 | 0.572 | 0.51-0.631 | 0.705 | 0.071 | 4.81e-01 |
| Aβ42 | 0.568 | 0.502-0.628 | 1.63 | 0.071 | 1.03e-01 |
| Aβ40/42 | 0.53 | 0.473-0.589 | 1.519 | 0.071 | 1.29e-01 |
| pTau181 | 0.493 | 0.432-0.549 | 0.045 | 0.071 | 9.64e-01 |
| NfL | 0.527 | 0.467-0.587 | 0.233 | 0.071 | 8.16e-01 |
| GFAP | 0.507 | 0.448-0.567 | 0.408 | 0.071 | 6.83e-01 |
| Clusterin | 0.489 | 0.429-0.549 | -1.002 | 0.072 | 3.17e-01 |
| CR1 | 0.515 | 0.458-0.579 | -0.25 | 0.072 | 8.02e-01 |
| C1q | 0.54 | 0.486-0.603 | -0.551 | 0.072 | 5.81e-01 |
| C1s | 0.526 | 0.466-0.587 | -1.537 | 0.072 | 1.24e-01 |
| Factor H | 0.561 | 0.502-0.62 | -0.909 | 0.072 | 3.63e-01 |

**Table S8: Results of Generalised Linear Models (GLMs) estimating protein levels from Age, Sex and an interaction term between phenotype (Control vs EOAD, Control vs LOAD, Control vs AD) and certain genotypes (SNP rsIDs).** Estimate values for each element (**Estimate**), the standard error (**Std Error**), the t-value statistic (**t-value**), significance thresholds (**Pr(>|t|)**) and formula used to create each GLM.

Early-Onset Alzheimer’s Disease

|  | Estimate | Std Error | t-value | Pr(>\|t\|) | Formula |
| --- | --- | --- | --- | --- | --- |
| (Intercept) | 92 | 34.2 | 2.69 | 0.00723 | Clusterin~Age+Sex+Phenotype(EOAD)*rs11136000 |
| Age | 1.27 | 0.411 | 3.09 | 0.00205 | Clusterin~Age+Sex+Phenotype(EOAD)*rs11136000 |
| SexM | -15.4 | 5.08 | -3.04 | 0.00247 | Clusterin~Age+Sex+Phenotype(EOAD)*rs11136000 |
| Phenotype1 | 62.5 | 11.6 | 5.37 | 9.9E-08 | Clusterin~Age+Sex+Phenotype(EOAD)*rs11136000 |
| rs11136000TC | 17 | 9.75 | 1.74 | 0.0816 | Clusterin~Age+Sex+Phenotype(EOAD)*rs11136000 |
| rs11136000TT | 7.66 | 13 | 0.591 | 0.555 | Clusterin~Age+Sex+Phenotype(EOAD)*rs11136000 |
| Phenotype1:rs11136000TC | -18.6 | 11.8 | -1.58 | 0.115 | Clusterin~Age+Sex+Phenotype(EOAD)*rs11136000 |
| Phenotype1:rs11136000TT | -6.3 | 16.1 | -0.392 | 0.695 | Clusterin~Age+Sex+Phenotype(EOAD)*rs11136000 |
| (Intercept) | 109 | 34.6 | 3.16 | 0.00164 | Clusterin~Age+Sex+Phenotype(EOAD)*rs9331888 |
| Age | 1.24 | 0.411 | 3.03 | 0.00254 | Clusterin~Age+Sex+Phenotype(EOAD)*rs9331888 |
| SexM | -15.9 | 5.07 | -3.13 | 0.00178 | Clusterin~Age+Sex+Phenotype(EOAD)*rs9331888 |
| Phenotype1 | 44 | 11 | 3.98 | 7.31E-05 | Clusterin~Age+Sex+Phenotype(EOAD)*rs9331888 |
| rs9331888GC | -7.27 | 9.09 | -0.799 | 0.424 | Clusterin~Age+Sex+Phenotype(EOAD)*rs9331888 |
| rs9331888GG | -26.1 | 17.3 | -1.51 | 0.133 | Clusterin~Age+Sex+Phenotype(EOAD)*rs9331888 |
| Phenotype1:rs9331888GC | 15.8 | 11.2 | 1.42 | 0.156 | Clusterin~Age+Sex+Phenotype(EOAD)*rs9331888 |
| Phenotype1:rs9331888GG | 19.1 | 20.8 | 0.915 | 0.36 | Clusterin~Age+Sex+Phenotype(EOAD)*rs9331888 |
| (Intercept) | 13.7 | 3.41 | 4.03 | 6.05E-05 | CR1~Age+Sex+Phenotype(EOAD)*rs6656401 |
| Age | 0.0457 | 0.0337 | 1.35 | 0.176 | CR1~Age+Sex+Phenotype(EOAD)*rs6656401 |
| SexM | -1.18 | 0.415 | -2.85 | 0.00454 | CR1~Age+Sex+Phenotype(EOAD)*rs6656401 |
| Phenotype1 | 2.51 | 2.53 | 0.992 | 0.321 | CR1~Age+Sex+Phenotype(EOAD)*rs6656401 |
| rs6656401AG | 0.964 | 2.18 | 0.443 | 0.658 | CR1~Age+Sex+Phenotype(EOAD)*rs6656401 |
| rs6656401GG | -1.39 | 2.11 | -0.66 | 0.51 | CR1~Age+Sex+Phenotype(EOAD)*rs6656401 |
| Phenotype1:rs6656401AG | -3.45 | 2.6 | -1.32 | 0.186 | CR1~Age+Sex+Phenotype(EOAD)*rs6656401 |
| Phenotype1:rs6656401GG | -2.49 | 2.53 | -0.985 | 0.325 | CR1~Age+Sex+Phenotype(EOAD)*rs6656401 |
| (Intercept)1 | 12 | 2.82 | 4.26 | 2.23E-05 | CR1~Age+Sex+Phenotype(EOAD)*rs6691117 |
| Age | 0.0632 | 0.0339 | 1.86 | 0.0629 | CR1~Age+Sex+Phenotype(EOAD)*rs6691117 |
| SexM | -1.18 | 0.419 | -2.81 | 0.00501 | CR1~Age+Sex+Phenotype(EOAD)*rs6691117 |
| Phenotype1 | -0.0958 | 0.828 | -0.116 | 0.908 | CR1~Age+Sex+Phenotype(EOAD)*rs6691117 |
| rs6691117GA | -1.19 | 0.811 | -1.46 | 0.144 | CR1~Age+Sex+Phenotype(EOAD)*rs6691117 |
| rs6691117GG | -1.64 | 1.86 | -0.881 | 0.378 | CR1~Age+Sex+Phenotype(EOAD)*rs6691117 |
| Phenotype1:rs6691117GA | 1.1 | 0.984 | 1.12 | 0.263 | CR1~Age+Sex+Phenotype(EOAD)*rs6691117 |
| Phenotype1:rs6691117GG | -0.822 | 2.61 | -0.314 | 0.753 | CR1~Age+Sex+Phenotype(EOAD)*rs6691117 |
| (Intercept) | 22.9 | 4.02 | 5.7 | 1.65E-08 | C1s~Age+Sex+Phenotype(EOAD)*rs3919533 |
| Age | 0.00304 | 0.0402 | 0.0758 | 0.94 | C1s~Age+Sex+Phenotype(EOAD)*rs3919533 |
| SexM | -1.18 | 0.496 | -2.37 | 0.0179 | C1s~Age+Sex+Phenotype(EOAD)*rs3919533 |
| Phenotype1 | 2.53 | 3 | 0.843 | 0.4 | C1s~Age+Sex+Phenotype(EOAD)*rs3919533 |
| rs3919533CT | 6 | 2.23 | 2.69 | 0.0072 | C1s~Age+Sex+Phenotype(EOAD)*rs3919533 |
| rs3919533TT | 5.8 | 2.13 | 2.73 | 0.00654 | C1s~Age+Sex+Phenotype(EOAD)*rs3919533 |
| Phenotype1:rs3919533CT | -4.47 | 3.05 | -1.47 | 0.143 | C1s~Age+Sex+Phenotype(EOAD)*rs3919533 |
| Phenotype1:rs3919533TT | -1.98 | 2.93 | -0.677 | 0.499 | C1s~Age+Sex+Phenotype(EOAD)*rs3919533 |
| (Intercept) | 398 | 55 | 7.23 | 1.04E-12 | FactorH~Age+Sex+Phenotype(EOAD)*rs6664877 |
| Age | -0.182 | 0.665 | -0.274 | 0.784 | FactorH~Age+Sex+Phenotype(EOAD)*rs6664877 |
| SexM | -18 | 8.2 | -2.19 | 0.0285 | FactorH~Age+Sex+Phenotype(EOAD)*rs6664877 |
| Phenotype1 | -41.8 | 16 | -2.61 | 0.00915 | FactorH~Age+Sex+Phenotype(EOAD)*rs6664877 |
| rs6664877 | 54.5 | 14.4 | 3.79 | 0.000159 | FactorH~Age+Sex+Phenotype(EOAD)*rs6664877 |
| Phenotype1:rs6664877 | -13.6 | 17.4 | -0.78 | 0.436 | FactorH~Age+Sex+Phenotype(EOAD)*rs6664877 |

Late-Onset Alzheimer’s Disease

|  | Estimate | Std Error | t-value | Pr(>\|t\|) | Formula |
| --- | --- | --- | --- | --- | --- |
| (Intercept) | 197 | 44.1 | 4.47 | 9.48E-06 | Clusterin~Age+Sex+Phenotype(LOAD)*rs11136000 |
| Age | 0.0545 | 0.532 | 0.102 | 0.918 | Clusterin~Age+Sex+Phenotype(LOAD)*rs11136000 |
| SexM | -14.5 | 6.71 | -2.16 | 0.0309 | Clusterin~Age+Sex+Phenotype(LOAD)*rs11136000 |
| Phenotype1 | 24.3 | 11.2 | 2.17 | 0.0305 | Clusterin~Age+Sex+Phenotype(LOAD)*rs11136000 |
| rs11136000TC | 4.4 | 10.4 | 0.423 | 0.672 | Clusterin~Age+Sex+Phenotype(LOAD)*rs11136000 |
| rs11136000TT | 3.39 | 14.8 | 0.229 | 0.819 | Clusterin~Age+Sex+Phenotype(LOAD)*rs11136000 |
| Phenotype1:rs11136000TC | -14.2 | 14.5 | -0.981 | 0.327 | Clusterin~Age+Sex+Phenotype(LOAD)*rs11136000 |
| Phenotype1:rs11136000TT | -5.44 | 20.8 | -0.261 | 0.794 | Clusterin~Age+Sex+Phenotype(LOAD)*rs11136000 |
| (Intercept) | 198 | 44.6 | 4.44 | 1.07E-05 | Clusterin~Age+Sex+Phenotype(LOAD)*rs9331888 |
| Age | 0.0724 | 0.533 | 0.136 | 0.892 | Clusterin~Age+Sex+Phenotype(LOAD)*rs9331888 |
| SexM | -14.7 | 6.7 | -2.2 | 0.028 | Clusterin~Age+Sex+Phenotype(LOAD)*rs9331888 |
| Phenotype1 | 16 | 10.4 | 1.54 | 0.124 | Clusterin~Age+Sex+Phenotype(LOAD)*rs9331888 |
| rs9331888GC | 1.35 | 9.91 | 0.136 | 0.892 | Clusterin~Age+Sex+Phenotype(LOAD)*rs9331888 |
| rs9331888GG | -3.77 | 17.3 | -0.217 | 0.828 | Clusterin~Age+Sex+Phenotype(LOAD)*rs9331888 |
| Phenotype1:rs9331888GC | -0.787 | 14.1 | -0.0559 | 0.955 | Clusterin~Age+Sex+Phenotype(LOAD)*rs9331888 |
| Phenotype1:rs9331888GG | 15.1 | 23.2 | 0.65 | 0.516 | Clusterin~Age+Sex+Phenotype(LOAD)*rs9331888 |
| (Intercept) | 13.6 | 4.4 | 3.1 | 0.00203 | CR1~Age+Sex+Phenotype(LOAD)*rs6656401 |
| Age | 0.0611 | 0.0448 | 1.36 | 0.173 | CR1~Age+Sex+Phenotype(LOAD)*rs6656401 |
| SexM | -0.992 | 0.559 | -1.77 | 0.0768 | CR1~Age+Sex+Phenotype(LOAD)*rs6656401 |
| Phenotype1 | 0.12 | 3.03 | 0.0398 | 0.968 | CR1~Age+Sex+Phenotype(LOAD)*rs6656401 |
| rs6656401AG | -1.3 | 2.54 | -0.511 | 0.61 | CR1~Age+Sex+Phenotype(LOAD)*rs6656401 |
| rs6656401GG | -2.6 | 2.48 | -1.05 | 0.294 | CR1~Age+Sex+Phenotype(LOAD)*rs6656401 |
| Phenotype1:rs6656401AG | -0.673 | 3.18 | -0.212 | 0.832 | CR1~Age+Sex+Phenotype(LOAD)*rs6656401 |
| Phenotype1:rs6656401GG | -0.0477 | 3.09 | -0.0154 | 0.988 | CR1~Age+Sex+Phenotype(LOAD)*rs6656401 |
| (Intercept) | 10.9 | 3.68 | 2.97 | 0.0031 | CR1~Age+Sex+Phenotype(LOAD)*rs6691117 |
| Age | 0.0721 | 0.0444 | 1.62 | 0.105 | CR1~Age+Sex+Phenotype(LOAD)*rs6691117 |
| SexM | -0.946 | 0.562 | -1.68 | 0.0928 | CR1~Age+Sex+Phenotype(LOAD)*rs6691117 |
| Phenotype1 | -0.189 | 0.747 | -0.253 | 0.801 | CR1~Age+Sex+Phenotype(LOAD)*rs6691117 |
| rs6691117GA | -1.21 | 0.87 | -1.39 | 0.164 | CR1~Age+Sex+Phenotype(LOAD)*rs6691117 |
| rs6691117GG | -0.106 | 2.13 | -0.0499 | 0.96 | CR1~Age+Sex+Phenotype(LOAD)*rs6691117 |
| Phenotype1:rs6691117GA | 1.02 | 1.23 | 0.833 | 0.405 | CR1~Age+Sex+Phenotype(LOAD)*rs6691117 |
| Phenotype1:rs6691117GG | -2.6 | 3.18 | -0.82 | 0.412 | CR1~Age+Sex+Phenotype(LOAD)*rs6691117 |
| (Intercept) | 18.6 | 4.87 | 3.81 | 0.000152 | C1s~Age+Sex+Phenotype(LOAD)*rs3919533 |
| Age | 0.0417 | 0.0493 | 0.846 | 0.398 | C1s~Age+Sex+Phenotype(LOAD)*rs3919533 |
| SexM | -0.731 | 0.62 | -1.18 | 0.239 | C1s~Age+Sex+Phenotype(LOAD)*rs3919533 |
| Phenotype1 | 6.45 | 3.73 | 1.73 | 0.0843 | C1s~Age+Sex+Phenotype(LOAD)*rs3919533 |
| rs3919533CT | 5.64 | 2.45 | 2.3 | 0.0218 | C1s~Age+Sex+Phenotype(LOAD)*rs3919533 |
| rs3919533TT | 7.27 | 2.37 | 3.07 | 0.00224 | C1s~Age+Sex+Phenotype(LOAD)*rs3919533 |
| Phenotype1:rs3919533CT | -7.78 | 3.87 | -2.01 | 0.045 | C1s~Age+Sex+Phenotype(LOAD)*rs3919533 |
| Phenotype1:rs3919533TT | -6.46 | 3.77 | -1.71 | 0.0871 | C1s~Age+Sex+Phenotype(LOAD)*rs3919533 |
| (Intercept) | 457 | 70.6 | 6.48 | 1.96E-10 | FactorH~Age+Sex+Phenotype(LOAD)*rs6664877 |
| Age | -0.884 | 0.852 | -1.04 | 0.3 | FactorH~Age+Sex+Phenotype(LOAD)*rs6664877 |
| SexM | -4.76 | 10.7 | -0.444 | 0.657 | FactorH~Age+Sex+Phenotype(LOAD)*rs6664877 |
| Phenotype1 | -46.6 | 13.8 | -3.37 | 0.000806 | FactorH~Age+Sex+Phenotype(LOAD)*rs6664877 |
| rs6664877 | 33.1 | 14.8 | 2.24 | 0.0257 | FactorH~Age+Sex+Phenotype(LOAD)*rs6664877 |
| Phenotype1:rs6664877 | 3.78 | 20.4 | 0.185 | 0.853 | FactorH~Age+Sex+Phenotype(LOAD)*rs6664877 |

Alzheimer’s Disease

|  | Estimate | Std Error | t-value | Pr(>\|t\|) | Formula |
| --- | --- | --- | --- | --- | --- |
| (Intercept) | 213 | 26.5 | 8.04 | 2.2E-15 | Clusterin~Age+Sex+Phenotype(AD)*rs11136000 |
| Age | -0.141 | 0.31 | -0.453 | 0.651 | Clusterin~Age+Sex+Phenotype(AD)*rs11136000 |
| SexM | -11.6 | 4.69 | -2.46 | 0.0139 | Clusterin~Age+Sex+Phenotype(AD)*rs11136000 |
| Phenotype1 | 27.8 | 9.9 | 2.8 | 0.00514 | Clusterin~Age+Sex+Phenotype(AD)*rs11136000 |
| rs11136000TC | 4.85 | 10.3 | 0.472 | 0.637 | Clusterin~Age+Sex+Phenotype(AD)*rs11136000 |
| rs11136000TT | 7.25 | 14.1 | 0.514 | 0.607 | Clusterin~Age+Sex+Phenotype(AD)*rs11136000 |
| Phenotype1:rs11136000TC | -7.07 | 11.8 | -0.599 | 0.549 | Clusterin~Age+Sex+Phenotype(AD)*rs11136000 |
| Phenotype1:rs11136000TT | -9.07 | 16.3 | -0.555 | 0.579 | Clusterin~Age+Sex+Phenotype(AD)*rs11136000 |
| (Intercept) | 221 | 26.5 | 8.32 | 2.43E-16 | Clusterin~Age+Sex+Phenotype(AD)*rs9331888 |
| Age | -0.14 | 0.31 | -0.451 | 0.652 | Clusterin~Age+Sex+Phenotype(AD)*rs9331888 |
| SexM | -11.6 | 4.69 | -2.48 | 0.0134 | Clusterin~Age+Sex+Phenotype(AD)*rs9331888 |
| Phenotype1 | 18.8 | 9.08 | 2.07 | 0.0385 | Clusterin~Age+Sex+Phenotype(AD)*rs9331888 |
| rs9331888GC | -8.58 | 9.75 | -0.88 | 0.379 | Clusterin~Age+Sex+Phenotype(AD)*rs9331888 |
| rs9331888GG | -7.42 | 17.9 | -0.415 | 0.678 | Clusterin~Age+Sex+Phenotype(AD)*rs9331888 |
| Phenotype1:rs9331888GC | 8.96 | 11.3 | 0.793 | 0.428 | Clusterin~Age+Sex+Phenotype(AD)*rs9331888 |
| Phenotype1:rs9331888GG | 5.82 | 20.2 | 0.289 | 0.773 | Clusterin~Age+Sex+Phenotype(AD)*rs9331888 |
| (Intercept) | 19.4 | 2.68 | 7.23 | 8.63E-13 | CR1~Age+Sex+Phenotype(AD)*rs6656401 |
| Age | 0.0115 | 0.023 | 0.501 | 0.617 | CR1~Age+Sex+Phenotype(AD)*rs6656401 |
| SexM | -1.03 | 0.346 | -2.98 | 0.00291 | CR1~Age+Sex+Phenotype(AD)*rs6656401 |
| Phenotype1 | -1.57 | 2.19 | -0.718 | 0.473 | CR1~Age+Sex+Phenotype(AD)*rs6656401 |
| rs6656401AG | -2.92 | 2.01 | -1.46 | 0.146 | CR1~Age+Sex+Phenotype(AD)*rs6656401 |
| rs6656401GG | -4.19 | 1.94 | -2.16 | 0.0311 | CR1~Age+Sex+Phenotype(AD)*rs6656401 |
| Phenotype1:rs6656401AG | 0.626 | 2.29 | 0.273 | 0.785 | CR1~Age+Sex+Phenotype(AD)*rs6656401 |
| Phenotype1:rs6656401GG | 1.11 | 2.22 | 0.501 | 0.617 | CR1~Age+Sex+Phenotype(AD)*rs6656401 |
| (Intercept) | 15.4 | 1.95 | 7.88 | 7.06E-15 | CR1~Age+Sex+Phenotype(AD)*rs6691117 |
| Age | 0.0186 | 0.023 | 0.806 | 0.42 | CR1~Age+Sex+Phenotype(AD)*rs6691117 |
| SexM | -1.02 | 0.348 | -2.93 | 0.00349 | CR1~Age+Sex+Phenotype(AD)*rs6691117 |
| Phenotype1 | -0.593 | 0.608 | -0.975 | 0.33 | CR1~Age+Sex+Phenotype(AD)*rs6691117 |
| rs6691117GA | -0.829 | 0.754 | -1.1 | 0.272 | CR1~Age+Sex+Phenotype(AD)*rs6691117 |
| rs6691117GG | -0.801 | 1.79 | -0.446 | 0.655 | CR1~Age+Sex+Phenotype(AD)*rs6691117 |
| Phenotype1:rs6691117GA | 0.36 | 0.868 | 0.415 | 0.678 | CR1~Age+Sex+Phenotype(AD)*rs6691117 |
| Phenotype1:rs6691117GG | -1.04 | 2.22 | -0.47 | 0.638 | CR1~Age+Sex+Phenotype(AD)*rs6691117 |
| (Intercept) | 19.7 | 3.54 | 5.58 | 2.93E-08 | C1s~Age+Sex+Phenotype(AD)*rs3919533 |
| Age | 0.0398 | 0.0294 | 1.35 | 0.176 | C1s~Age+Sex+Phenotype(AD)*rs3919533 |
| SexM | -0.952 | 0.444 | -2.14 | 0.0324 | C1s~Age+Sex+Phenotype(AD)*rs3919533 |
| Phenotype1 | 4.47 | 3.09 | 1.45 | 0.148 | C1s~Age+Sex+Phenotype(AD)*rs3919533 |
| rs3919533CT | 5.47 | 2.58 | 2.12 | 0.0342 | C1s~Age+Sex+Phenotype(AD)*rs3919533 |
| rs3919533TT | 6.1 | 2.5 | 2.45 | 0.0146 | C1s~Age+Sex+Phenotype(AD)*rs3919533 |
| Phenotype1:rs3919533CT | -5.52 | 3.19 | -1.73 | 0.0838 | C1s~Age+Sex+Phenotype(AD)*rs3919533 |
| Phenotype1:rs3919533TT | -3.72 | 3.1 | -1.2 | 0.23 | C1s~Age+Sex+Phenotype(AD)*rs3919533 |
| (Intercept) | 337 | 39 | 8.66 | 1.49E-17 | FactorH~Age+Sex+Phenotype(AD)*rs6664877 |
| Age | 0.538 | 0.464 | 1.16 | 0.246 | FactorH~Age+Sex+Phenotype(AD)*rs6664877 |
| SexM | -17.7 | 7.01 | -2.53 | 0.0116 | FactorH~Age+Sex+Phenotype(AD)*rs6664877 |
| Phenotype1 | -24.4 | 11.7 | -2.09 | 0.0368 | FactorH~Age+Sex+Phenotype(AD)*rs6664877 |
| rs6664877 | 47.3 | 13.5 | 3.51 | 0.000459 | FactorH~Age+Sex+Phenotype(AD)*rs6664877 |
| Phenotype1:rs6664877 | -17.9 | 15.7 | -1.15 | 0.252 | FactorH~Age+Sex+Phenotype(AD)*rs6664877 |

 **Figure S1.** Forest plot of regression coefficients (estimate values) of the generalised linear models (**A** = early-onset Alzheimer’s disease (EOAD), **B** = late-onset Alzheimer’s disease (LOAD)), showing the direction and strength of effect for ATN biomarkers and complement proteins measured for each AD group vs. controls. Proteins with negative coefficients are shown in **green** and those with a positive coefficient are shown in **red**. Proteins shown in **grey** were not statistically significant at the Bonferroni corrected p-value significance thresholds (* = p<0.005; ** = p<0.001; *** = p<0.0001). Error bars indicate the standard error for each regression coefficient.

B

A


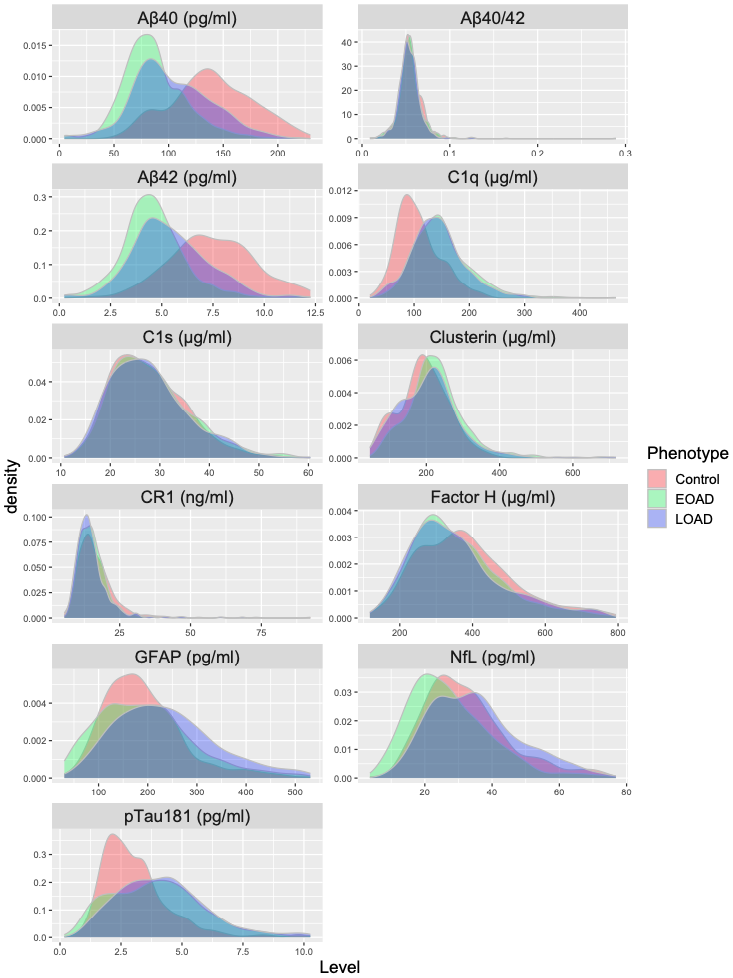


**Figure S2.** Density plots showing distributions of plasma protein levels (each protein labelled below the X-axes) for each of the groups (**red** = controls, **green** = early-onset Alzheimer’s disease (EOAD), **blue** = late-onset Alzheimer’s disease (LOAD)).


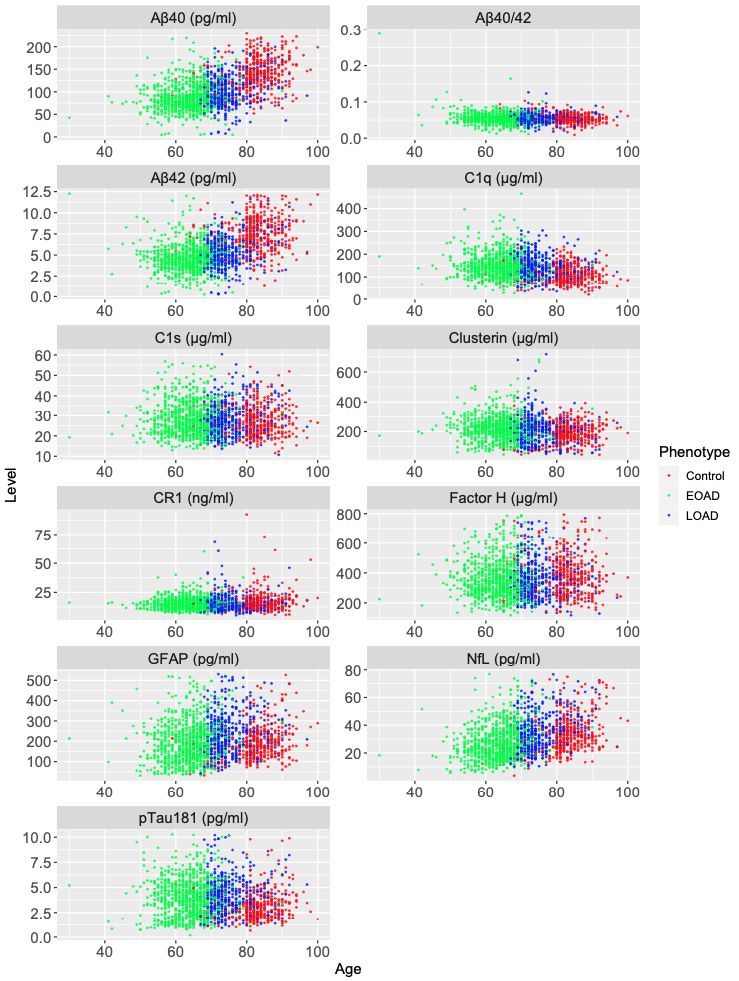


**Figure S3.** Scatter plots showing distributions of plasma protein levels on the Y axes (each protein labelled on the Y-axes) vs age at inclusion on the X-axes for each of the groups (**red** = controls, **green** = early-onset Alzheimer’s disease (EOAD), **blue** = late-onset Alzheimer’s disease (LOAD)).
